# N6-adenosine-methyltransferase MTA70-like participates antiviral responses in *Nicotiana benthamiana*

**DOI:** 10.3389/fmicb.2025.1716357

**Published:** 2026-01-08

**Authors:** Haijuan Wang, Tianyi Zhang, Jiaxin Xu, Zhenqi Sun, Zhaoran Wu, Baolong Zhang, Qianqian Zhao, Xiangdong Yang, Mingmin Zhao

**Affiliations:** 1 College of Horticulture and Plant Protection, Inner Mongolia Agricultural University, Hohhot, China; 2 Jilin Academy of Agricultural Sciences, Changchun, China; 3 Key Laboratory of the Development and Resource Utilization of Biological Pesticide in Inner Mongolia, Hohhot, China

**Keywords:** N6-adenosine-methyltransferase MTA70-like, ToMTA70, *Nicotiana benthamiana*, Tomato, potato virus Y, antiviral responses

## Abstract

**Introduction:**

Previous studies have shown that m6A methylation has a certain regulatory role in the replication of human viruses and plant viruses. Particularly, the m6A methylation in the host endogenous gene significantly decreased after the infection of potato virus Y (PVY).

**Methods:**

Here, we cloned and characterized N6-adenosine-methyltransferase MTA70-like (ToMTA70) from tomato. The ToMTA70 was constructed into the pMDC32 binary vector. To create the ToMTA70 mutant, sequence of the MTA70 superfamily domain (493-653) with PDS and GUS sequences, respectively. The effect of the ToMTA70 on viral accumulation was studied using an Agrobacterium-mediated transient expression assay.

**Results:**

A conserved MT-A70 superfamily domain in ToMTA70 was identified by encoding sequence and structural analyses, which indicating ToMTA70 might involve in m6A methylation. The transient expression of ToMTA70 inhibited viral accumulation of PVY. When the conserved domain of MTA70 super family was replaced by PDS and GUS (ToMTA70-PDS or ToMTA70-GUS), the PVY infection was increased in plants comparing with the control. However, transiently expression of ToMTA70 did not show any influence on TVMV and TMV infection.

**Discussion:**

This suggests that ToMTA70 participate in plant antiviral responses of PVY, which can be applied for engineering the anti-viral strategy.

## 1 Introduction

N6-methyladenosine (m6A) is the most abundant mRNA modification, which plays diverse RNA-regulatory roles in transcript processing and translation. Apart from messenger RNAs (mRNAs), transfer RNAs (tRNAs), ribosomal RNAs (rRNAs), small non-coding RNAs, and long non-coding RNAs (lncRNAs) could be modified by m6A (Roundtree et al., 2017; Liu et al., 2016; Zhao et al., 2017; Nachtergaele and He, 2017). Several studies have identified plant m6A to be dynamically regulated, controlling plant development and defense response (Zhong et al., 2008; Wan et al., 2015; Zhang et al., 2019). It has been demonstrated that there is a strong association between m6A and cancers in human system (Li et al., 2024; Shen et al., 2021). In plants, m6A methylation modification also regulates virus infection and plays a key role in plant-virus interactions. It has been showed that the m6A level is reduced upon Tobacco mosaic virus (TMV) (Li et al., 2018), Plum pox virus (PPV) and Potato virus Y (PVY) (Yue et al., 2022).

m6A methylation is also involving in viral infection of Alfalfa mosaic virus (AMV). The viral accumulation and movement are inhibited by the deletion of the m6A demethylase gene at ALKBH9B in *Arabidopsis thaliana*. However, this is not the case of Cucumber mosaic virus (CMV) (Martínez-Pérez et al., 2017, 2021). Down-regulation of *N. benthamiana* AlkB homologs of the plant-specific ALKBH9 clade caused a significant decrease in PPV and PVY accumulation. This provides experimental evidences that supports the m6A implication in Potyvirus infection (Yue et al., 2022).

METTLs is protein family whose members play key roles in the methylation of DNA and RNA molecules (Wong and Eirin-Lopez, 2021). In mammals, the main components of the m6A-MTase complex include METTL3, METTL14, Wilms' tumor 1-associating protein (WTAP) and KIAA0823. METTL3 is the SAM-binding subunit, which is highly conserved in eukaryotes and whose deletion in mice leads to early embryonic death (Geula et al., 2015). Several studies nonetheless showed that cellular METTLs affect viral infection of eukaryotic hosts. METTL3 and METTL14 is associated with human immune deficiency virus 1 (HIV-1) replication (Lichinchi et al., 2016). m6A modification of hepatitis C virus (HCV) RNA is mediated by METTL3 and METTL14, and regulated by WTAP, which showed a negative role of m6A in HCV infection (Gokhale et al., 2016). MTase domains recur in diverse viral taxa, but their contribution in m6A deposition and cellular epitranscriptomic dynamics is currently unknown. T-DNA insertion disruption of *Arabidopsis thaliana* MTA (TAIR: AT4G10760), an METTL3 homolog, causes an embryo lethal phenotype (Zhong et al., 2008). MTB (TAIR: AT4G09980) is an METTL14 homolog shown to be a component of the *A. thaliana* complex responsible for m6A deposition in mRNAs (RuŽička et al., 2017).

Recently, it has been reported that transcription factor NFYA3_0 positively regulates the transcription of the m6A methyltransferase gene NbMTA. Loss-of-function similarly compromises resistance to the virus while diminishing m6A abundance (Li et al., 2025). NbMTA methylated the PVY coat protein-coding region, which facilitating viral RNA degradation and effectively restricting the viral infection. These findings suggest that the core antiviral mechanism of nuclear transcription factor NFYA3_0 realizes the targeted degradation of viral RNA by activating the methyltransferase NbMTA-mediated m6A epitranscriptome regulation.

Here, we identified ToMTA70 (a homolog of MTA) from tomato. The sequence and protein characteristics of ToMTA70 were analyzed. The effect of ToMTA70 on viral infection of PVY was studied in *N. benthamian*.

## 2 Materials and methods

### 2.1 Plant materials and viral inoculation

*N. benthamiana* and Tomato plants were grown in a greenhouse at 22~24°C under a 16/8 h light/dark cycle. PVY-Ros1 infection clone (PVY carrying the Rosea1 gene) was constructed and reported by Professor Jose Antonio Daros from Spain (Cordero et al., 2017). The PVY-Ros1 extract was mechanically inoculated to *N. benthamiana* plants. The upper systemic leaves were collected and stored at−80°C, and used as the inoculum source. PVY-Ros1 inoculum preparation in phosphate buffer and mechanical inoculation were done as described (Pasin et al., 2014). The infection clone of TVMV (Zhao et al., 2020)and TMV-GFP (Casper and Holt, 1996) were previously described.

### 2.2 The ToMTA70 cloning

The 1g of leaves for total RNA was purified from tomato leaves by Trizol reagent. Then, 1ug total RNA was used for cDNA reactions by Evo M-MLV RT Mix Kit. Specific primers were designed according to the MTA70 sequence (XM_004245125.4) (Supplementary Table S1). PCR reaction is 50 μL, including primers 2 μL, Prime STAR GXL Premix (2x) 25 μL, cDNA 1 μL. Pre-denaturation reaction at 95°C for 5 min, 33 PCR cycles (98°C for 15 s, 60°C for 15 s, 68°C for 2 min25s), and 72°C for 10 min. The PCR products, named as ToMTA70, were run in 1% agarose gel. The agar-gel DNA purification was used to recover and purify PCR fragments. The products were inserted into the pMD19-T plasmid to obtain pMD19-T-ToMTA70 and transformed into *E. coli* DH5a. Then the plasmid was subjected to sequence. The ToMTA70 was cloned into pDONR207 using the BP recombination reaction to create pDONR207-ToMTA70 and verified by *Xba* Idigestion (Supplementary Figure S4). The ToMTA70 was cloned into the pMDC32 binary vector using the LR reaction to obtain pMDC32-ToMTA70 and verified by *Pst* Idigestion (Supplementary Figure S4).

To create the ToMTA70 mutant, sequence of the MTA70 superfamily domain (493–653) with PDS and GUS sequences, respectively. A specific fragment (11813 bp) was amplified from the plasmid pMDC32-ToMTA70 by primers of pMDC32_GUS-F/R and pMDC32_PDS-F/R (Supplementary Table S1). The 673 bp and 340 bp fragments were amplified from pTRV2-GUS and pTRV2-PDS using the primers 70-GUS_F/R and 70-PDS_F/R (Supplementary Table S1). The PCR amplification products were recombined using one-step Gibson assembly ligation to obtain pMDC32-ToMTA70-PDS(ToMTA70-PDS) and pMDC32-ToMTA70-GUS(ToMTA70-GUS). PCR amplification with 340 bp and 673 bp was used to confirm the positive clones (Supplementary Figure S6). Sanger sequencing of ToMTA70-PDS and ToMTA70-GUS plasmids showed that mutant nucleotides were successfully introduced (Supplementary Figures S7, S8).

### 2.3 Transient expression assay

The tested plasmids were transformed into Agrobacterium strain C58C1 by the freeze-thaw method (Pasin, 2022). *Agrobacterium* strains carrying the ToMTA70 expression vectors were cultured overnight at 28°C. Cultures were then centrifuged at 4000 rpm at room temperature (5 min), the supernatant was discarded and bacterial pellets were suspended at OD_600_ = 1. Then, the pellets were suspended in induction buffer containing 0.5 M 2-(N-Morpholino) ethanesulfonic acid hydrate (pH 5.6), 1 M MgCl2 and 0.1 M acetosyringone. Bacterial suspensions were incubated at room temperature (3 h), and then used to infiltrate leaves of *N. benthamiana* plants (4–6 leaves) using a needleless syringe. Samples from leaves infiltrated with the empty pMDC32 vector were used as a control.

### 2.4 *In silico* sequence and structure analysis of ToMTA70 protein

Sequences alignment of ToMTA70 was done by ESPript3 (https://espript.ibcp.fr/ESPript/cgi-bin/ESPript.cgi). ProtParam (https://web.expasy.org/protparam/) was used for analysis of the physicochemical properties of ToMTA70. NovoPro (https://www.novopro.cn/tools/?source=chemnav.net) was used for analysis of secondary structures of ToMTA70. ScanProsite tool (https://prosite.expasy.org/scanprosite/) was used for *in silico* analyses of conserved domains of the ToMTA70. Three-dimensional structural models of ToMTA70 was predicted by Swiss-Model(https://swissmodel.expasy.org/interactive). The structural similarity matrix between ToMTA70 and different species was obtained by TBtools. The structural tree diagram between different species was obtained using MEGA11 software.

### 2.5 RT-qPCR analysis

Total RNA was purified and used in cDNA synthesis reactions. Then, cDNA aliquots were used in PCR reactions performed with SYBR Green Premix Pro Taq HS qPCR Kit (Rox PlusPlus) and gene-specific primers (Supplementary Table S1) in an FTC-3000P Real-Time Quantitative Thermal Cycler (Funglyn Biotech, Canada). Expression was normalized using NbUBI as a reference, and fold changes relative to the control condition were calculated by the ΔΔCT method.

### 2.6 Western blot

Viral accumulation of PVY-Ros1 was determined by western blot analysis as described (Wang et al., 2025). Total protein extracts from plant samples were prepared. The proteins were separated by SDS-PAGE electrophoresis and transferred onto PVDF membranes. The Geldoc image system (Thermo Fisher Scientific, USA) was used to take pictures of the protein gel and used as the loading control. Immunodetection was conducted using primary antibodies of anti-CP(PVY), anti-CP(TVMV) and anti-GFP for detecting corresponding viruses. Horseradish peroxidase-conjugated goat anti-rabbit immunoglobulin (IgG; ab205718; Abcam) were used as secondary antibodies. Protein signals were visualized by enhanced chemiluminescence. Quantitative values of the immunoblot assay were normalized to the sum of replicate methods.

### 2.7 m6A methylation detection

m6A methylation detection was done with the EpiQuik™ m6A RNA Methylation Quantification Kit (Colorimetric) (EpigenTek Group Inc.) as previously described (Yue et al., 2022). The total RNA is bound to strip wells using RNA high binding solution. m6A is detected using capture and detection antibodies. The detected signal is enhanced and then quantified colorimetrically by reading the absorbance in a microplate spectrophotometer. To determine the relative m6A RNA methylation status of two different RNA samples, a simple calculation for the percentage of m6A in total RNA was carried out using the formula.

## 3 Results

### 3.1 ToMTA70 contains a conserved domain of MTA70 superfamily in several plant species

**Figure 1:**
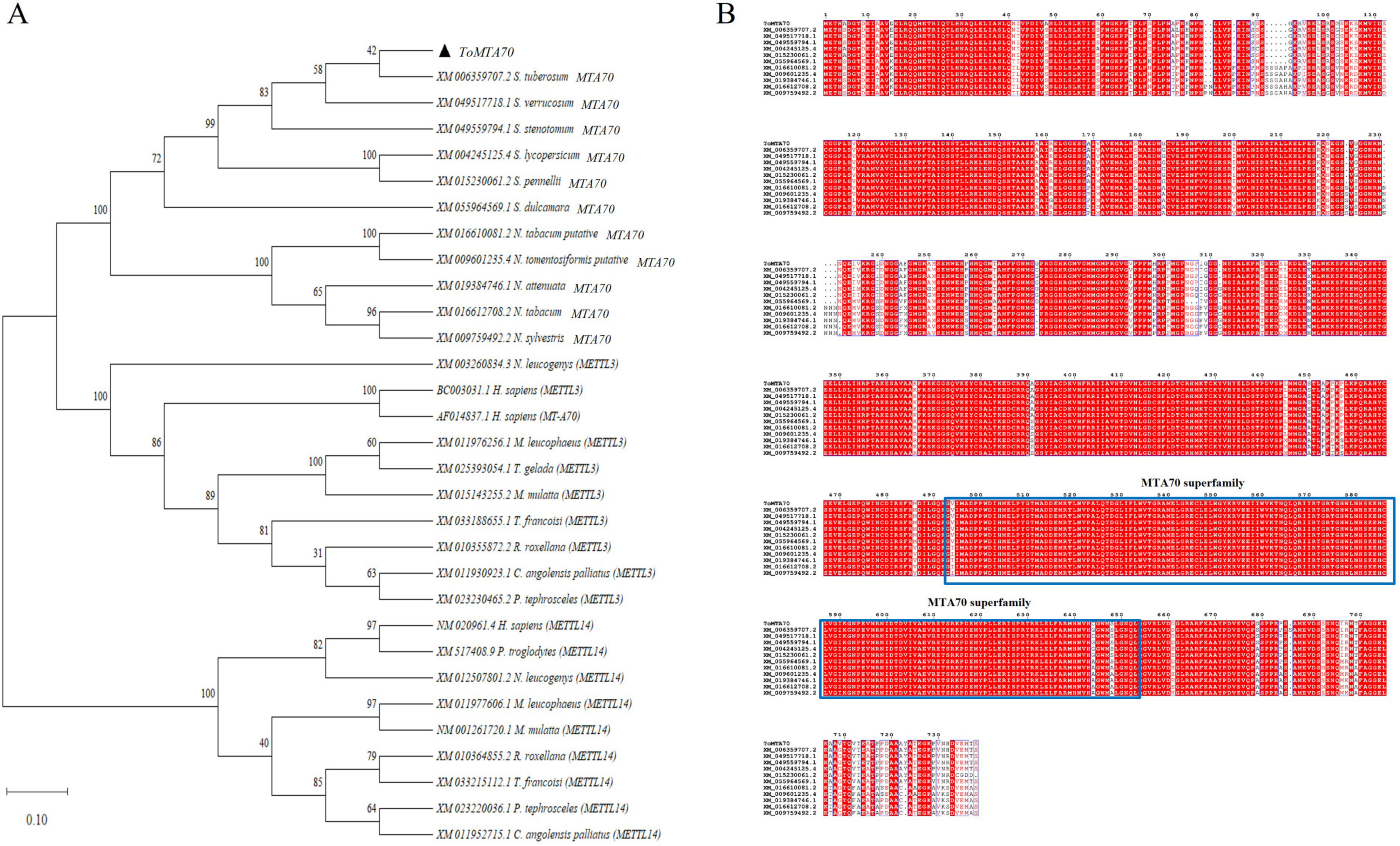
Phylogenetic and alignment analysis of ToMTA70. **(A)** The phylogenetic tree was constructed based on the ToMTA70 nucleotide sequences, METTL3 and METTL14 using the neighbor-joining method in MEGA11 software. The ToMTA70 is labeled with a black arrow. **(B)** Alignment of protein sequence of ToMTA70 by ESPript3. The conserved domain of MTA70 superfamily was labeled with blue square.

ToMTA70 (2217 bp) was obtained from tomato by PCR (Supplementary Figure S1 and Supplementary Table S1) and sequenced (Supplementary Figures S2, S3). The sequence was subjected to a BLAST search against public databases of METTL3, METTL14 and MTA70-like (Figure 1A). we found that ToMTA70 is closed to a gene of *S. tuberosum* (XM006359707.2). Next, amino acid sequence alignment of ToMTA70 was performed against different plant species by ESPript3 (Figure 1B). A conserved MT-A70 superfamily domain (493–653 aa) in ToMTA70 was identified by encoding sequence and structural analyses (Supplementary Figure S5), which indicating ToMTA70 might involve in m6A methylation.

### 3.2 In silico sequence analysis of ToMTA70

738 amino acids of ToMTA70 was obtained after translation (Supplementary Table S2). The physicochemical properties of ToMTA70 protein was analyzed by ProtParam webserver software (Supplementary Table S3). The molecular formula of ToMTA70 protein is C_3531_H_5687_N_1029_O_1081_S_48_. The molecular weight of ToMTA70 is 81 kDa. The isoelectric point of ToMTA70 is 6.37 and belongs to hydrophilic protein. Based on the protein signature database analyzed by InterPro, we identified the N6-adenosine-methyltransferase MT-A70 protein as belonging to the S-adenosine-L-methionine dependent methyltransferase superfamily.

**Figure 2:**
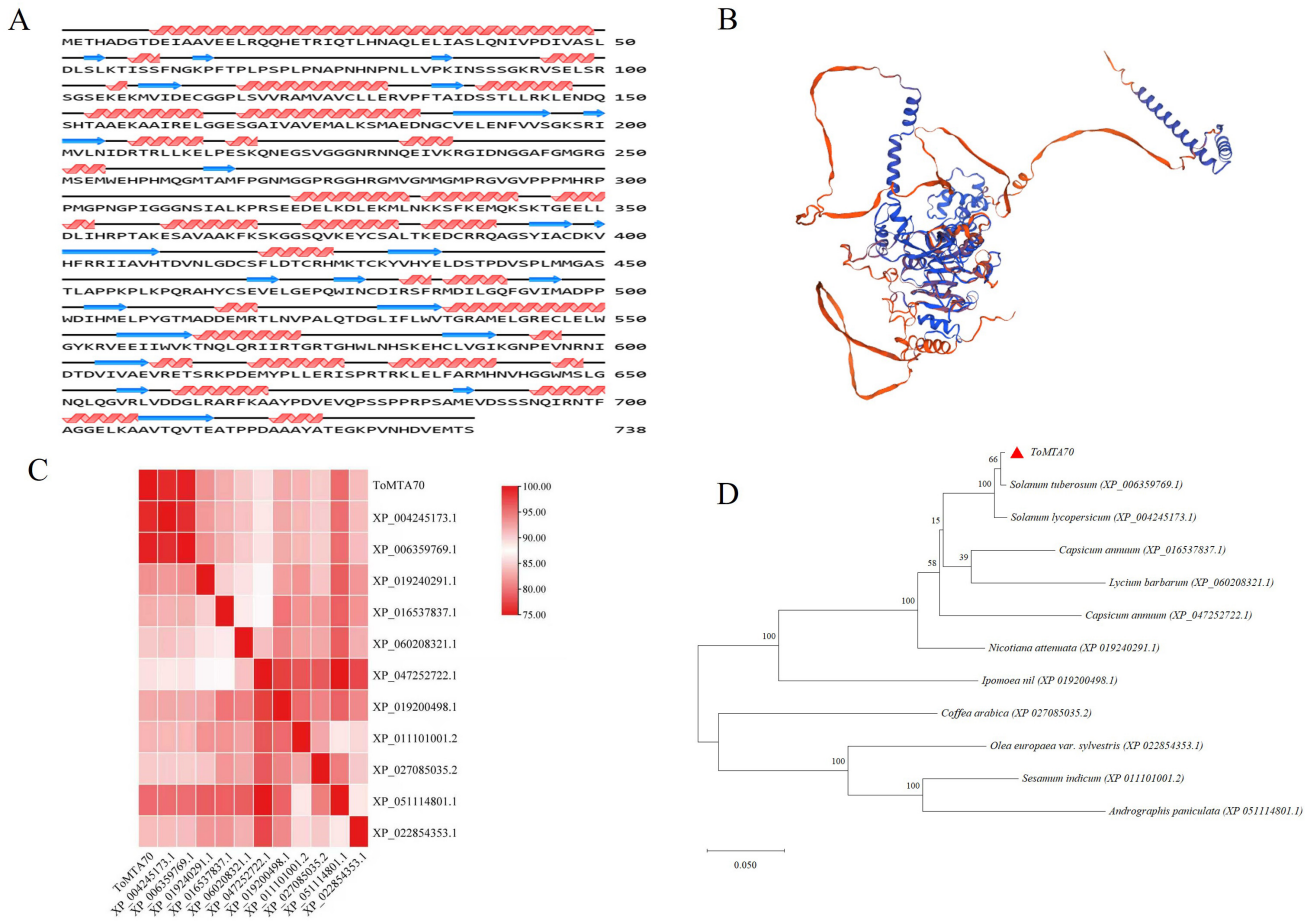
The prediction and chanracteristic of ToMTA70 protein. **(A)** The secondary structure of ToMTA70 protein predicted by NovoPro. Blue arrows indicate alpha helixes, and these arrows are distributed along the sequence, showing the location and approximate extent of the α-helix in this protein sequence. The red tilde represents the β-folded structure. **(B)** The tertiary structure of ToMTA70 protein predicted by Swiss-Model. **(C)** The structural similarity matrix of ToMTA70 predicted by TBtools. Colors represets Dali Z-scores. **(D)** The protein blast of ToMTA70 with other plant species by MEGA 11. Branch support values are shown.

The secondary structure of ToMTA70 was analyzed using NovoPro, which including 24 alpha helixes and β-fold structures (Figure 2A). The tertiary structure of ToMTA70 with a GMQE value of 0.72 was predicted by Swiss-Model (Figure 2B). Next, we identified the ToMTA70 was closed to *Solaum tuberosum* (XP 006359769.1) from the structural similarity matrix predicted by TBtools (Figure 2C and Supplementary Table S4) and the protein blast of ToMTA70 by MEGA 11(Figure 2D and Supplementary Table S4).

### 3.3 Transiently expression of ToMTA70 inhibits viral infection of PVY in *N. benthamiana*

**Figure 3:**
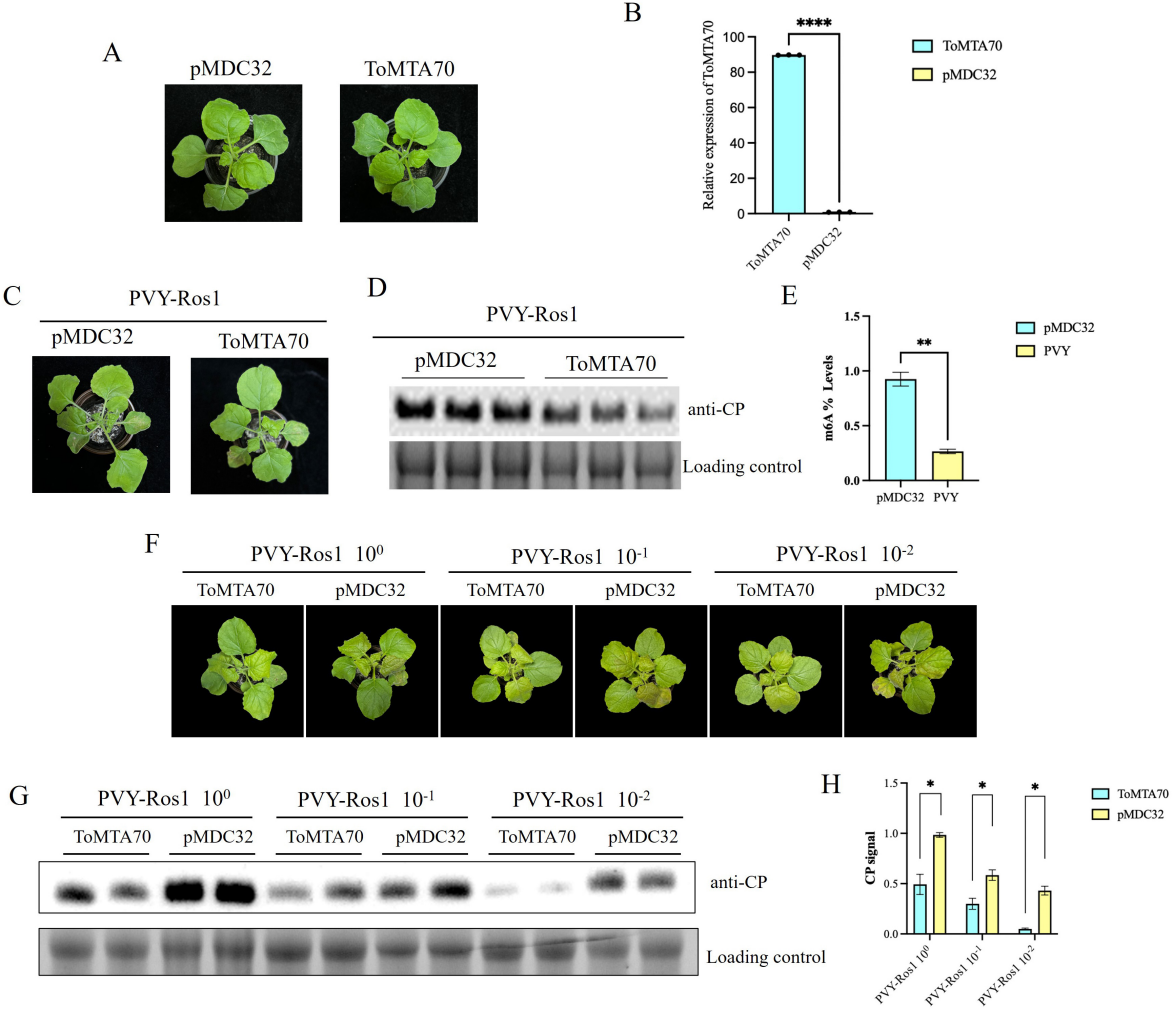
Effect of ToMTA70 on PVY-Ros1 virus. **(A)** Plant phenotype in *N. benthamiana* after transient expression of ToMTA70 protein at 3 dpi. **(B)** ToMTA70 expression in agro-infiltrated leaves was detected by RT-qPCR. Quantity ONE was used to quantify ToMTA70 protein signal. **(C)** The viral infection syptoms of PVY-Ros1 in plants transciently expressed with ToMTA70 and pMDC32 were photographed at 9 dpi. **(D)** Viral accumulation in upper uninoculated systemic leaves was measured by Western blot with an anti-CP serum of PVY. RuBisCO large subunit is shown as a loading control. **(E)** m6A amount in total RNA samples was quantified by EpiQuiK m6A RNA methylation Quantification Kit. Values are plotted as mean ± standard deviation. Staitstic analysis was done by Student's t test. **** represents significant difference at *p* < 0.0001. ** represents *p* < 0.01. **(F)** Crude extracts (100)of PVY-Ros1infected *N. benthamiana* leaves were dilluted to 10–1 and 10–2. Then, the infiltrated leaves of ToMTA70 were inoculated with the dilluted extracts. At 9 dpi, the viral infection syptoms of PVY-Ros1 in plants were photographed. **(G)** Viral accumulation of PVY-Ros1was detected by Western blot with anti-CP. **(H)** Quantification of CP signals are shown. Values are plotted as mean ± standard deviation. Staitstic analysis was done by Student's t test. ns represents no significant.* represents difference at *p* < 0.05. Three biological replicates were used.

To verify the influence of ToMTA70 on viral infection, the plasmid pMDC32-ToMTA70 (ToMTA70) was constructed by gateway recombination reaction (Supplementary Figure S4). The pMDC32-ToMTA70 was transformed into *Agrobacterium* C58C1 and infiltrated into *N. benthamiana*. At 3 dpi, there was no significant differences in plant phenotype compared with control pMDC32 (Figure 3A). RT-qPCR was used to detect the expression level of ToMTA70 in infiltrated plants (Figure 3B). At 3 days later, crude extract of PVY-Ros1 was inoculated on the infiltrated leaves. Viral symptoms were observed at 9 dpi and showed clearly attenuated (Figure 3C). Virus accumulation of PVY-Ros1in the upper leaves was analyzed by Western blotting. We found PVY-Ros1 accumulation was significantly reduced in plants transiently expression of ToMTA70 compared with the control (Figure 3D). The m6A methylation of the upper non-inoculated leaves was detected by Colorimetric assay. The results showed that m6A methylation of PVY-Ros1 infected plants significantly decreased comparing with those of pMDC32 (Figure 3E). Further, the ToMTA70-infiltrated plants were inoculated with the diluted PVY-Ros1 extracts (10^0^, 10^−1^, and 10^−2^). We found that with the decreasing concentration of PVY-Ros1 extracts, the viral accumulation were significant decreased in ToMTA70-infiltrated plants and pMDC32-infiltared plants (*p* < 0.05) (Figures 3F–H). This suggests that transiently expression of ToMTA70 inhibits viral infection of PVY in *N. benthamiana*.

### 3.4 The upregulation of PVY-Ros1 infection in ToMTA70 mutant treated plants

**Figure 4:**
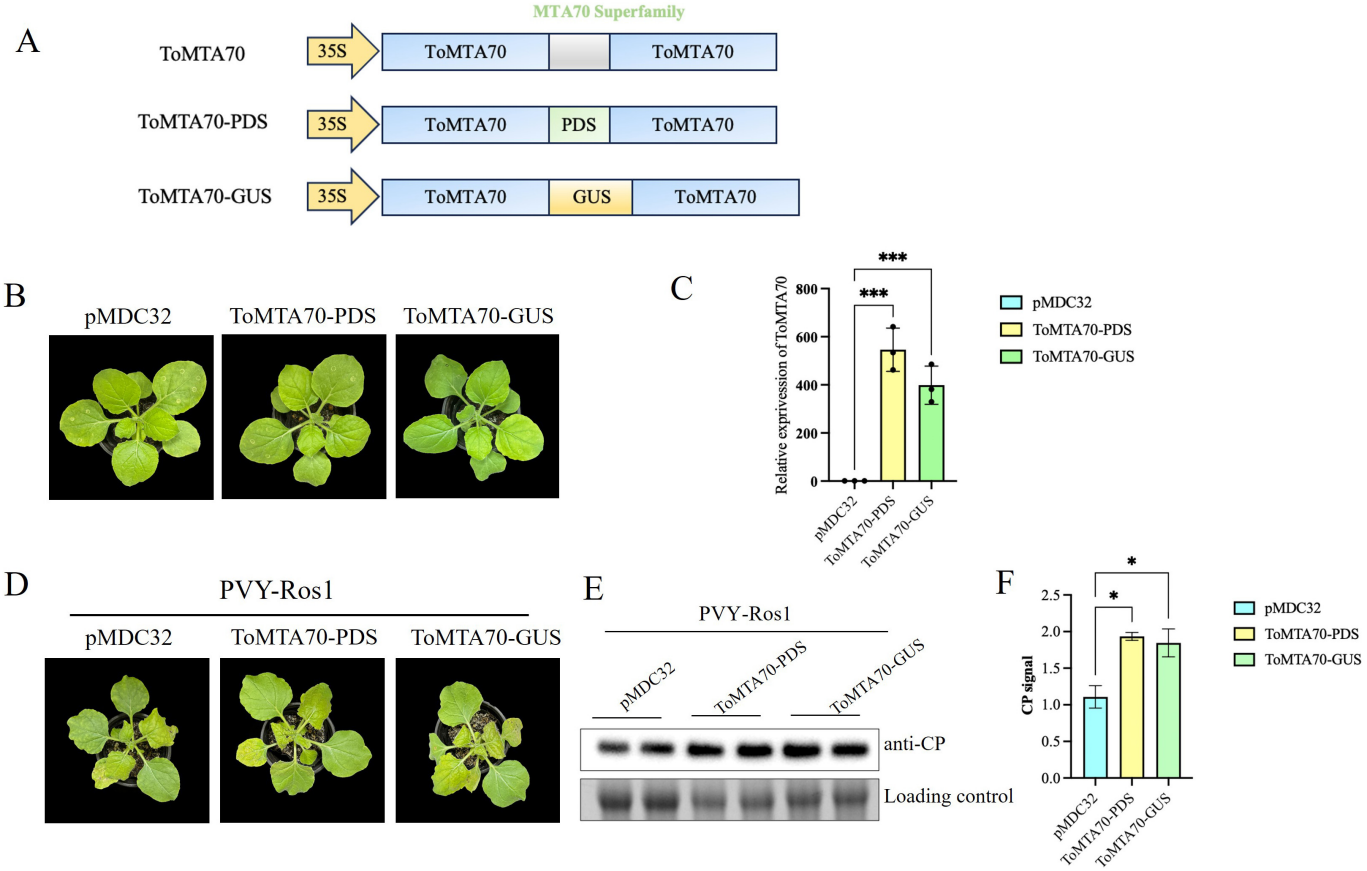
Effect of transient expression ToMTA70 mutants on PVY-Ros1 infection in *N. benthamiana*. **(A)** The schemetic of ToMTA70 mutants constuction. The conserved domain of MTA70 superfamily in ToMTA70 was replaced by PDS and GUS to create ToMTA70-PDS and ToMTA70-GUS. **(B)** Plant phenotype of *N. benthamiana* infiltrated with ToMTA70-PDS, ToMTA70-GUS and pMDC32 at 3 dpi. **(C)** The express of ToMTA70 in plants infiltrated with ToMTA70-PDS, ToMTA70-GUS and pMDC32 was detected by RT-qPCR at 3 dpi. **(D)** The viral infection syptoms of PVY-Ros1 in plants transciently expressed with ToMTA70-PDS, ToMTA70-GUS and pMDC32 were photographed at 9 dpi. **(E)** Viral accumulation in upper uninoculated systemic leaves was measured by Western blot with an anti-CP serum of PVY. RuBisCO large subunit is shown as a loading control. **(F)** Quantification of CP signals are shown. Values are plotted as mean ± standard deviation. Staitstic analysis was done by Student's t test. *** represents significant difference at *p* < 0.001. * represents *p* < 0.05.

We created the mutant constructs to verify the antiviral effect of ToMTA70. The conserved domain (MTA superfamily, 493–653) of ToMTA70 were replaced by PDS or GUS sequences to obtain pMDC32-ToMTA70-PDS (ToMTA70-PDS) and pMDC32-ToMTA70-GUS (ToMTA70-GUS) (Figure 4A). Then, the plasmids were transformed into *Agrobacterium* C58C1 and infiltrated into *N. benthamiana*. No significant phenotype differences in treated plants compared with control were observed (Figure 4B). The high expression of ToMTA70-PDS and ToMTA70-GUS in *N. benthamiana* was detected by RT-qPCR (Figure 4C). The infiltrated plants were inoculated by PVY-Ros1 extract, at 9 dpi, we found that the viral infection symptom in plants treated by ToMTA70-PDS and ToMTA70-GUS became sever comparing the control (Figure 4D). Correspondingly, the viral accumulation of PVY-Ros1 are significant increasing than that of the control (*p* < 0.05) (Figures 4E, F). This is supporting that ToMTA70 inhibits PVY-Ros1 infection.

### 3.5 Effect of transiently expression ToMTA70 on TVMV and TMV infection in *N. benthamiana*

**Figure 5:**
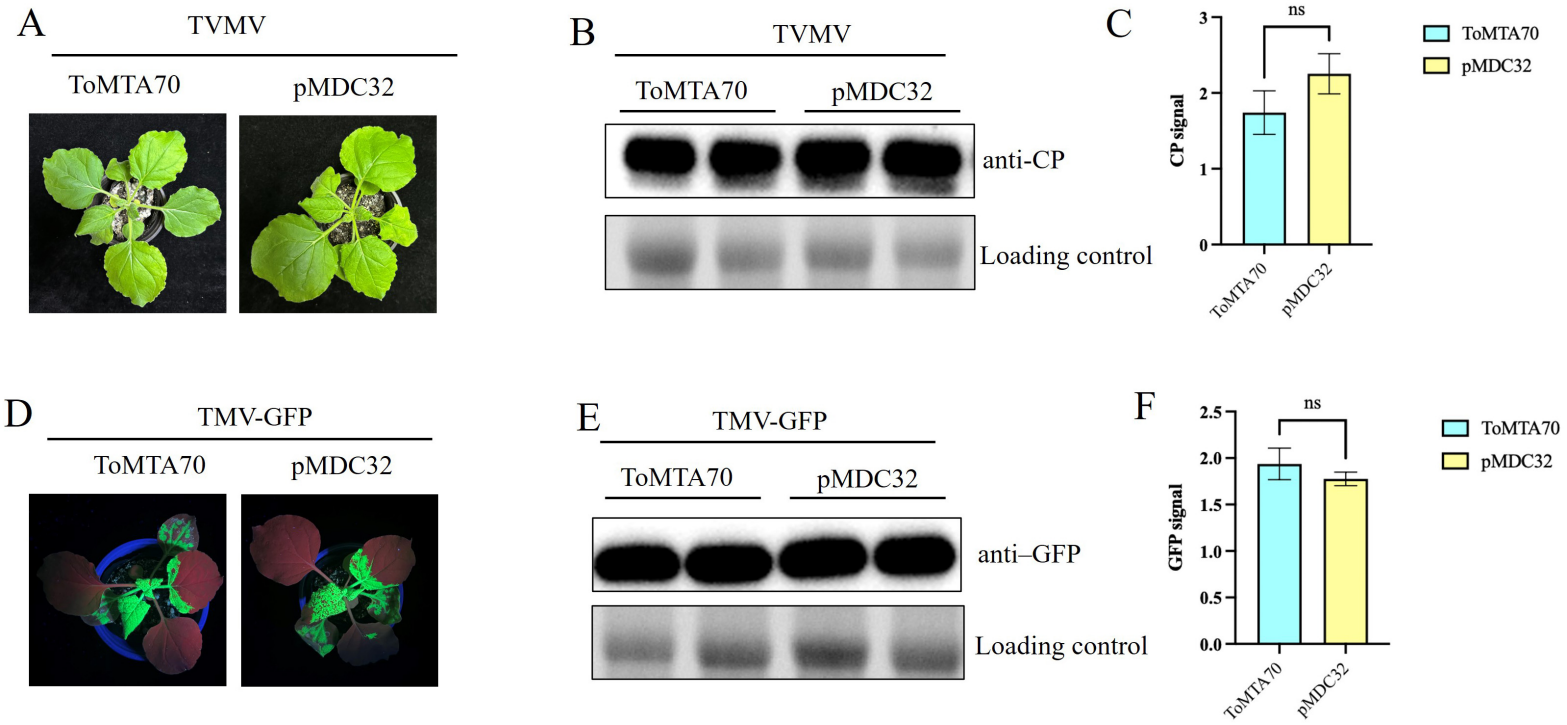
Effect of ToMTA70 on the other virus. **(A)** The viral infection syptoms of TVMV in plants transciently expressed with ToMTA70 and pMDC32 were photographed at 9 dpi. **(B)** Viral accumulation in upper uninoculated systemic leaves was measured by Western blot with an anti-CP serum of TVMV. RuBisCO large subunit is shown as a loading control. **(C)** Quantification of CP signals are shown. **(D)** The viral infection syptoms of TMV in plants transciently expressed with ToMTA70 and pMDC32 was photographed at 5 dpi. **(E)** Viral accumulation in upper uninoculated systemic leaves was measured by Western blot with an anti-GFP serum. RuBisCO large subunit is shown as a loading control. **(F)** Quantification of GFP signals are shown. Values are plotted as mean ± standard deviation. Staitstic analysis was done by Student's t test. ns represents no significant.

To verify the influence of ToMTA70 on other viral infection, the infection clone TVMV and TMV-GFP were co-infiltrated with pMDC32-ToMTA70 and empty vector pMDC32 in *N. benthamiana* plants. The results showed no significant differences in viral symptoms of TVMV (at 9 dpi) (Figure 5A) and TMV-GFP (at 5 dpi) (Figure 5D). According to the results of western blot and quantification of CP or GFP signals, the viral accumulation of TVMV and TMV-GFP in ToMTA70-treated plants and the control were similar (Figures 5B, C, E, F). This suggests that ToMTA70 has not influence on TVMV and TMV infection.

## 4 Discussion

In plants, m6A functionally modulates a variety of processes including development and responses to biotic and abiotic stress (Wei et al., 2023; Li et al., 2023). In plants, the core element of the methyltransferase complex is MTA, which functions to bind the methyl donor S-adenosyl methionine (SAM) and transfer the methyl group to the target adenylate for modification. Notably, studies have demonstrated that the homozygous mutant of MTA produces an embryonic lethal phenotype (Liu et al., 2014). In rice, transcriptome analysis of the Mta ABI3: MTA revealed that a large number of down-regulated genes are involved in transport or targeted transport processes, whereas most of the up-regulated genes are associated with stress and stimulus response pathways. OsMTA2 belongs to a part of rice methyltransferase, which affects m6A methylation, and then affects rice panicle length, pollination rate and effective seed number (Zhang et al., 2019). Over expression of PtrMTA promoted the root development and showed stronger drought stress tolerance (Lu et al., 2020). These findings indicate that MTA is involved in plant development and adaptation to environmental stresses. In *A. thaliana*, AtMTA is required for m6A methylation and related with embryo development (Zhong et al., 2008). In this study, we identified ToMTA70 from tomato plant, which is similar to *Nicotiana attenuata* (XP019240291.l). The coding sequence of ToMTA70 contains a conserved domain of MTA superfamily. The tertiary structure of ToMTA70 is similar to that of l1MKT1_SOYBN gene in *soybean*.

We cloned ToMTA70 into binary vector and conducted the transient expression assay. The results showed that plant phenotype did not show any difference comparing with the control.

When inoculated with PVY-Ros1 in ToMTA70-infiltrated plants, viral accumulation showed significantly decreased. This is indicating that ToMTA70 could inhibit the PVY infection. To further confirm this function of ToMTA70 in PVY infection, we create the ToMTA70 mutants, ToMTA70-PDS and ToMTA70-GUS. We found that transiently expression of ToMTA70 mutants could result in increasing of PVY infection. However, ToMTA70 showed no influence on TMV and TVMV infection. In this case, we speculated that ToMTA70 might particularly recognize PVY RNA and exert the methylation role.

## Data Availability

The raw data supporting the conclusions of this article will be made available by the authors, without undue reservation.

## References

[B1] Casper S. J. Holt C. A. (1996). Expression of the green fluorescent protein-encoding gene from a tobacco mosaic virus-based vector. Gene 173, 69–73. doi: 10.1016/0378-1119(95)00782-2 8707059

[B2] Cordero T. Mohamed M. A. López-Moya J. J. Daròs J. A. (2017). A recombinant Potato virus Y infectious clone tagged with the Rosea1 visual marker (PVY-Ros1) facilitates the analysis of viral infectivity and allows the production of large amounts of anthocyanins in plants. Front. Microbiol. 8, 611. doi: 10.3389/fmicb.2017.00611 28428782 PMC5382215

[B3] Geula S. Moshitch-Moshkovitz S. Dominissini D. Mansour A. A. Kol N. Salmon-Divon M. . (2015). Stem cells. m6A mRNA methylation facilitates resolution of naïve pluripotency toward differentiation. Science 347, 1002–1006. doi: 10.1126/science.1261417 25569111

[B4] Gokhale N. S. McIntyre A. B. R. McFadden M. J. Roder A. E. Kennedy E. M. Gandara J. A. . (2016). N6-Methyladenosine in Flaviviridae viral RNA genomes regulates infection. Cell Host Microbe 20, 654–665. doi: 10.1016/j.chom.2016.09.015 27773535 PMC5123813

[B5] Li H. Jiang Y. Chen J. Li Z. Zhang R. Wei 2Y. . (2024). Systematic characterization of m6A proteomics across 12 cancer types: a multi-omics integration study. Mol. Omics 20, 103–114. doi: 10.1039/d3mo00171g 37942799

[B6] LiJ. LuoJ. HeH. WangF. WuH. ZhaoC. . (2025). The nuclear transcription factor NFYA3_0 promotes MTA-mediated m6A modification of Potato virus Y genomic RNA to confer antiviral resistance in Nicotiana benthamiana. Plant Commun. 101584. doi: 10.1016/j.xplc.2025.10158441176619

[B7] Li J. Wang J. Pang Q. Yan X. (2023). Analysis of N6-methyladenosine reveals a new important mechanism regulating the salt tolerance of sugar beet (Beta vulgaris). Plant Sci. 335:111794. doi: 10.1016/j.plantsci.2023.111794 37459955

[B8] Li Z. Shi J. Yu L. Zhao X. Ran L. Hu D. . (2018). N 6 -methyl-adenosine level in Nicotiana tabacum is associated with tobacco mosaic virus. Virol. J. 15:87. doi: 10.1186/s12985-018-0997-4 29769081 PMC5956853

[B9] Lichinchi G. Gao S. Saletore Y. Gonzalez G. M. Bansal V. Wang Y. . (2016). Dynamics of the human and viral m(6)A RNA methylomes during HIV-1 infection of T cells. Nat. Microbiol. 1:16011. doi: 10.1038/nmicrobiol.2016.111 27572442 PMC6053355

[B10] Liu J. Yue Y. Han D. Wang X. Fu Y. Zhang L. . (2014). A METTL3-METTL14 complex mediates mammalian nuclear RNA N6-adenosine methylation. Nat. Chem. Biol. 10, 93–95. doi: 10.1038/nchembio.1432 24316715 PMC3911877

[B11] Liu J. Zhu Y. Luo G. Z. Wang X. Yue Y. Wang X. . (2016). Abundant DNA 6mA methylation during early embryogenesis of zebrafish and pig. Nat. Commun. 7:13052. doi: 10.1038/ncomms13052 27713410 PMC5059759

[B12] Lu L. Zhang Y. He Q. Qi Z. Zhang G. Xu W. . (2020). MTA, an RNA m6A methyltransferase, enhances drought tolerance by regulating the development of trichomes and roots in poplar. Int. J. Mol. Sci. 21:2462. doi: 10.3390/ijms21072462 32252292 PMC7177244

[B13] Martínez-Pérez M. Aparicio F. López-Gresa M. P. Bellés J. M. Sánchez-Navarro J. A. Pallás V. (2017). Arabidopsis m6A demethylase activity modulates viral infection of a plant virus and the m6A abundance in its genomic RNAs. Proc. Natl. Acad. Sci. U.S.A. 114, 10755–10760. doi: 10.1073/pnas.1703139114 28923956 PMC5635872

[B14] Martínez-Pérez M. Gómez-Mena C. Alvarado-Marchena L. Nadi R. Micol J. L. Pallas V. . (2021). The m6A RNA demethylase ALKBH9B plays a critical role for vascular movement of alfalfa mosaic virus in Arabidopsis. Front. Microbiol. 12:745576. doi: 10.3389/fmicb.2021.745576 34671333 PMC8521051

[B15] Nachtergaele S. He C. (2017). The emerging biology of RNA post-transcriptional modifications. RNA Biol. 14, 156–163. doi: 10.1080/15476286.2016.1267096 27937535 PMC5324755

[B16] Pasin F. (2022). Assembly of plant virus agroinfectious clones using biological material or DNA synthesis. STAR Protoc. 3:101716. doi: 10.1016/j.xpro.2022.101716 36149792 PMC9519601

[B17] Pasin F. Simón-Mateo C. García J. A. (2014). The hypervariable amino-terminus of P1 protease modulates potyviral replication and host defense responses. PLoS Pathog. 10:e1003985. doi: 10.1371/journal.ppat.1003985 24603811 PMC3946448

[B18] Roundtree I. A. Luo G. Z. Zhang Z. Wang X. Zhou T. Cui Y. . (2017). YTHDC1 mediates nuclear export of N6-methyladenosine methylated mRNAs. eLife 6:e31311. doi: 10.7554/eLife.31311 28984244 PMC5648532

[B19] RuŽička K. Zhang M. Campilho A. Bodi Z. Kashif M. Saleh M. . (2017). Identification of factors required for m6 A mRNA methylation in Arabidopsis reveals a role for the conserved E3 ubiquitin ligase HAKAI. New Phytol. 215, 157–172. doi: 10.1111/nph.14586 28503769 PMC5488176

[B20] Shen S. Zhang R. Jiang Y. Li Y. Lin L. Liu Z. . (2021). Comprehensive analyses of m6A regulators and interactive coding and non-coding RNAs across 32 cancer types. Mol. Cancer 20:67. doi: 10.1186/s12943-021-01362-2 33849552 PMC8045265

[B21] Wan Y. Tang K. Zhang D. Xie S. Zhu X. Wang Z. . (2015). Transcriptome-wide high-throughput deep m(6)A-seq reveals unique differential m(6)A methylation patterns between three organs in Arabidopsis thaliana. Genome Biol. 16:272. doi: 10.1186/s13059-015-0839-2 26667818 PMC4714525

[B22] Wang H. Zhang J. Meng Z. Sun Z. Liu D. Li B. . (2025). Cu/Zn superoxide dismutase homologs participate in Nicotiana benthamiana antiviral responses. Front. Microbiol. 16:1561731. doi: 10.3389/fmicb.2025.1561731 40756210 PMC12313585

[B23] Wei J. Li H. Gui Y. Zhou H. Zhang R. Zhu K. . (2023). Coordination of m6A mRNA methylation and gene transcriptome in sugarcane response to drought stress. Plants 12:3668. doi: 10.3390/plants12213668 37960025 PMC10650135

[B24] Wong J. M. Eirin-Lopez J. M. (2021). Evolution of Methyltransferase-Like (METTL) proteins in Metazoa: a complex gene family involved in epitranscriptomic regulation and other epigenetic processes. Mol. Biol. Evol. 38, 5309–5327. doi: 10.1093/molbev/msab267 34480573 PMC8662637

[B25] Yue J. Wei Y. Sun Z. Chen Y. Wei X. Wang H. . (2022). AlkB RNA demethylase homologues and N6 -methyladenosine are involved in Potyvirus infection. Mol. Plant Pathol. 23, 1555–1564. doi: 10.1111/mpp.13239 35700092 PMC9452765

[B26] Zhang F. Zhang Y. C. Liao J. Y. Yu Y. Zhou Y. F. Feng Y. Z. . (2019). The subunit of RNA N6-methyladenosine methyltransferase OsFIP regulates early degeneration of microspores in rice. PLoS Genet. 15:e1008120. doi: 10.1371/journal.pgen.1008120 31116744 PMC6548400

[B27] Zhao B. S. Roundtree I. A. He C. (2017). Post-transcriptional gene regulation by mRNA modifications. Nat. Rev. Mol. Cell Biol. 18, 31–42. doi: 10.1038/nrm.2016.132 27808276 PMC5167638

[B28] Zhao M. García B. Gallo A. Tzanetakis I. E. Simón-Mateo C. García J. A. . (2020). Home-made enzymatic premix and Illumina sequencing allow for one-step Gibson assembly and verification of virus infectious clones. Phytopathol. Res. 2:36. doi: 10.1186/s42483-020-00077-4 33768973 PMC7990137

[B29] Zhong S. Li H. Bodi Z. Button J. Vespa L. Herzog M. . (2008). MTA is an Arabidopsis messenger RNA adenosine methylase and interacts with a homolog of a sex-specific splicing factor. Plant Cell 20, 1278–1288. doi: 10.1105/tpc.108.058883 18505803 PMC2438467

