# Supplementary material for: N6-adenosine-methyltransferase MTA70-like participates antiviral responses in Nicotiana benthamiana
Source: Front Microbiol. 2026 Jan 8;16:1716357. doi: 10.3389/fmicb.2025.1716357 (PMC12826071; doi:10.3389/fmicb.2025.1716357)

**Figure S1 Amplification and construction of T-ToMTA70**

(A) *ToMTA70* fragments obtained by PCR with primer ToMTA70_F/R in agarose gel.

(B) *ToMTA70* fragments were amplified by PCR in *E. coli* DH5a.


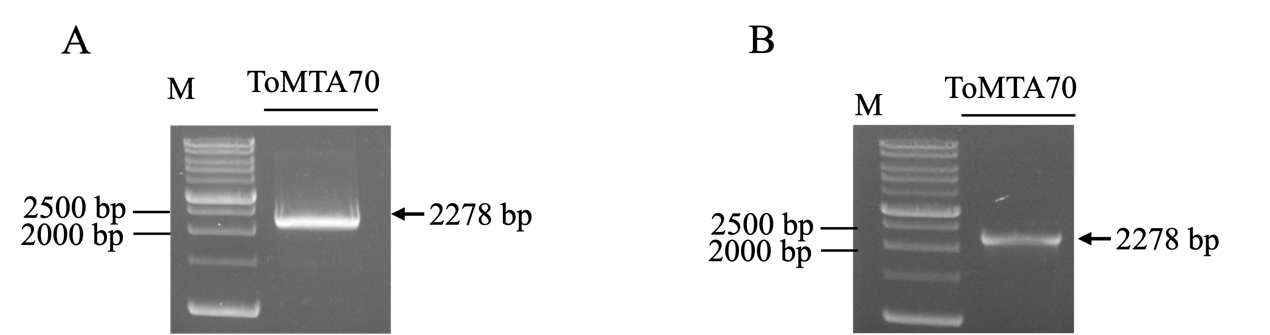


**Figures S2 Sequence of ToMTA70**

ATGGAAACTCATGCTGACGGCACAGATGAAATCGCCGCCGTGGAGGAGCTCCGGCAACAGCACGAAACCCGTATACAAACCCTACACAACGCTCAATTGGAACTCATCGCCTCACTACAAAACATAGTTCCTGATATAGTCGCTTCACTCGACCTCTCTCTTAAGACCATTTCATCCTTCAATGGAAAACCCTTCACCCCTTTACCCAGCCCACTCCCTAACGCCCCCAACCATAACCCTAATTTACTCGTCCCAAAGATCAATTCCAGTTCTGGTAAACGTGTTTCAGAACTTTCTAGAAGTGGTAGTGAGAAGGAGAAAATGGTAATTGATGAATGTGGGGGACCTTTGTCGGTTGTAAGGGCGATGGTTGCAGTTTGTTTGTTAGAGAGAGTGCCGTTTACTGCTATTGATTCGTCTACTTTGTTAAGGAAGTTAGAGAATGATCAGTCGCATACGGCTGCTGAAAAGGCAGCGATTAGGGAGCTCGGAGGGGAGTCGGGGGCGATAGTGGCAGTGGAAATGGCATTGAAATCGATGGCGGAGGATAATGGTTGTGTAGAATTGGAGAATTTTGTGGTTAGCGGGAAATCTAGGATTATGGTGTTGAATATTGATAGAACTAGGCTATTGAAAGAATTGCCGGAAAGTAAGCAGAATGAGGGAAGTGTTGGTGGAGGAAACCGGAATAATCAGGAAATAGTGAAAAGAGGGATTGATAATGGAGGAGCTTTTGGAATGGGAAGGGCAATGAGTGAAATGTGGGAGCATCCACATATGCAGGGGATGACAGCTATGTTTCCCGGGAATATGGGCGGTCCAAGAGGAGGGCATAGAGGAATGGTGGGTATGATGGGGATGCCGAGAGGTGTTGGCGTTCCACCTCCTATGCATAGACCACCAATGGGGCCAAATGGGCCCATAGGAGGAGGGAATTCTATTGCTTTAAAACCTAGGTCAGAAGAAGATGAGCTGAAGGATTTAGAGAAAATGTTGAATAAGAAAAGTTTTAAGGAAATGCAGAAATCAAAAACCGGAGAGGAGTTATTGGACCTTATTCATCGTCCCACTGCGAAGGAATCTGCTGTCGCTGCAAAGTTCAAAAGCAAAGGTGGTTCCCAGGTGAAGGAATATTGTTCAGCTTTAACAAAGGAAGATTGTCGACGCCAAGCTGGTTCCTACATTGCTTGTGATAAGGTTCATTTTCGGCGAATAATTGCTGTGCATACTGATGTCAACTTGGGTGATTGCTCTTTTCTTGATACTTGCCGTCACATGAAGACATGCAAGTATGTACATTATGAGCTTGACTCGACGCCAGATGTATCACCCCTGATGATGGGAGCATCTACCTTGGCCCCTCCCAAGCCTTTGAAACCTCAGCGTGCTCATTACTGTTCAGAAGTAGAGCTTGGTGAACCACAATGGATTAATTGTGACATACGGTCATTTAGAATGGATATTTTAGGGCAGTTTGGAGTTATAATGGCTGATCCACCATGGGATATTCATATGGAATTGCCTTATGGGACAATGGCTGATGATGAAATGCGCACTTTAAATGTCCCTGCACTGCAAACTGATGGTCTTATATTCCTTTGGGTCACCGGACGTGCAATGGAGCTTGGACGGGAATGTCTAGAGCTTTGGGGTTACAAGCGTGTTGAGGAGATTATTTGGGTTAAGACCAATCAACTTCAGCGAATCATCAGAACTGGACGGACAGGCCATTGGCTCAATCACAGCAAGGAACATTGCCTTGTTGGAATAAAGGGAAATCCAGAGGTGAACAGGAATATTGATACTGATGTCATAGTAGCAGAGGTCCGGGAAACAAGTCGTAAGCCAGATGAGATGTACCCCTTGCTCGAAAGAATTAGTCCAAGGACAAGAAAGCTGGAGTTATTTGCGCGGATGCACAATGTTCATGGCGGGTGGATGTCACTTGGAAACCAACTACAAGGGGTACGATTAGTTGATGACGGGCTTAGGGCGCGGTTCAAGGCTGCATATCCTGATGTGGAGGTGCAACCTTCATCACCTCCAAGGCCATCTGCAATGGAAGTAGATTCAAGTTCTAATCAAATAAGGAATACATTTGCTGGAGGGGAATTAAAGGCTGCAGTGACCCAGGTCACTGAGGCTACACCCCCAGATGCAGCAGCCTATGCAACTGAAGGAAAACCAGTAAACCATGATGTGGAGATGACTAGCTAA

**Figures S3 Sequence map of ToMTA70**


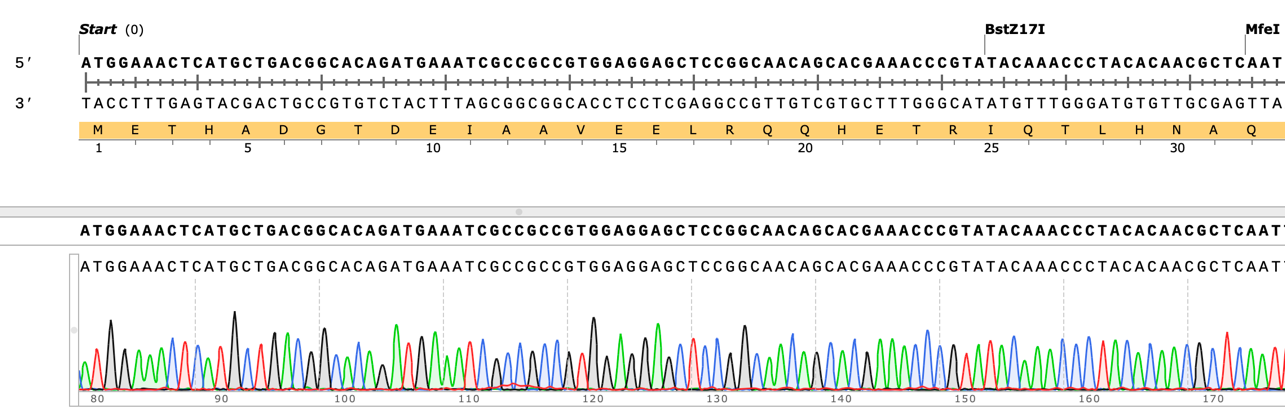


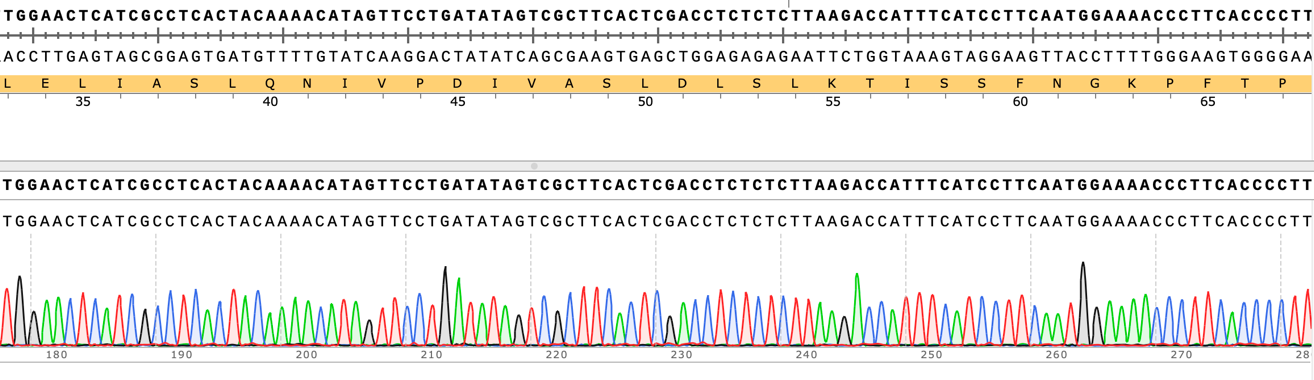


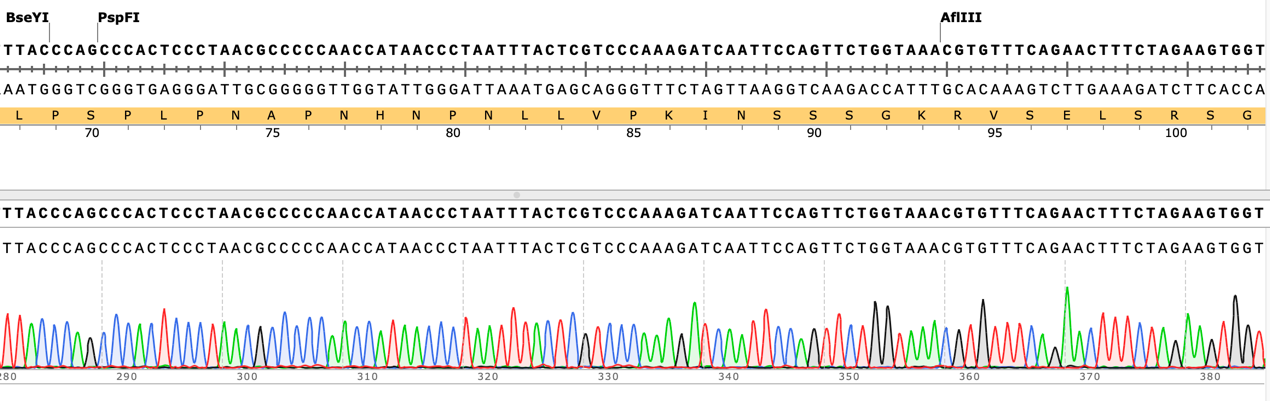


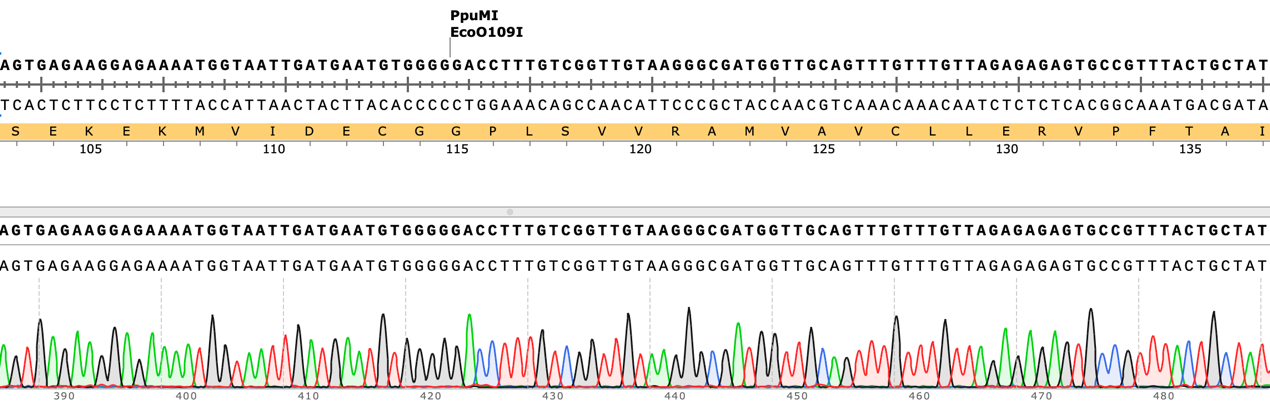


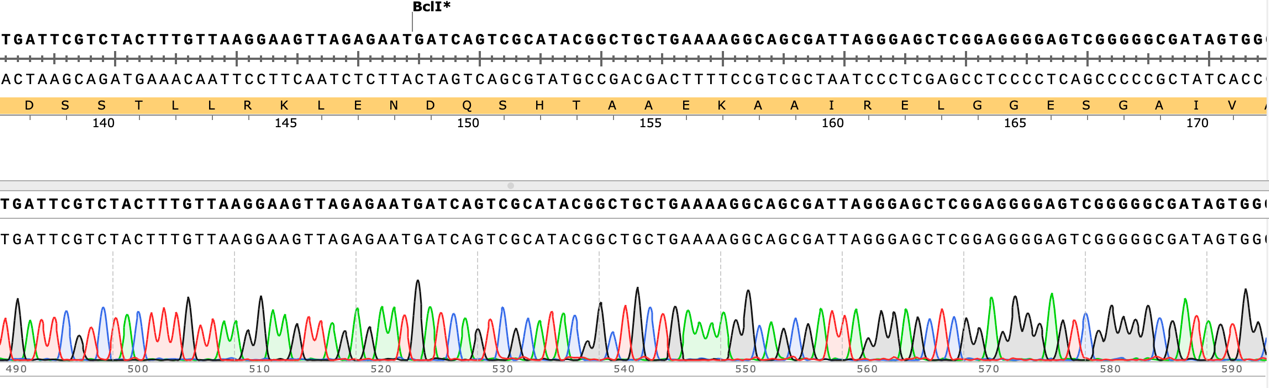


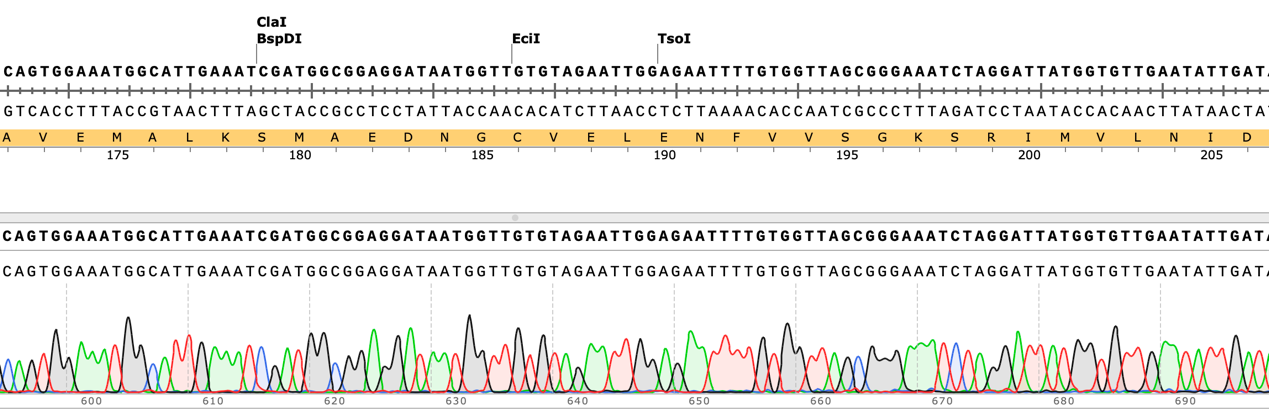


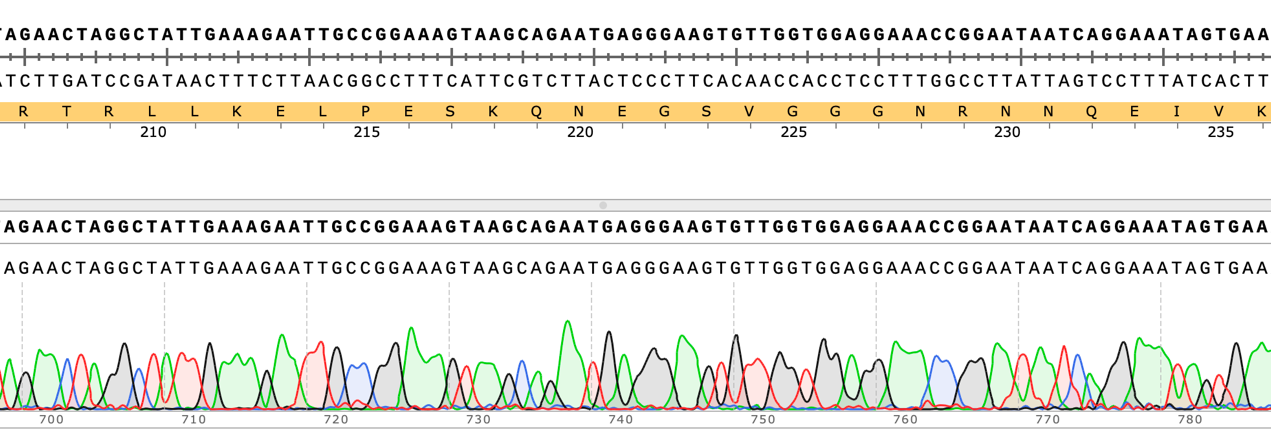


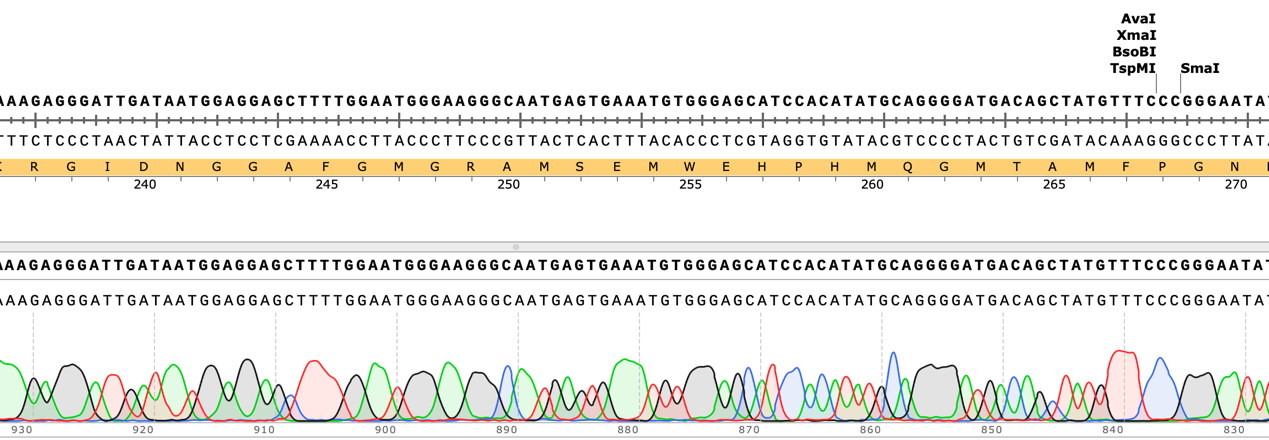


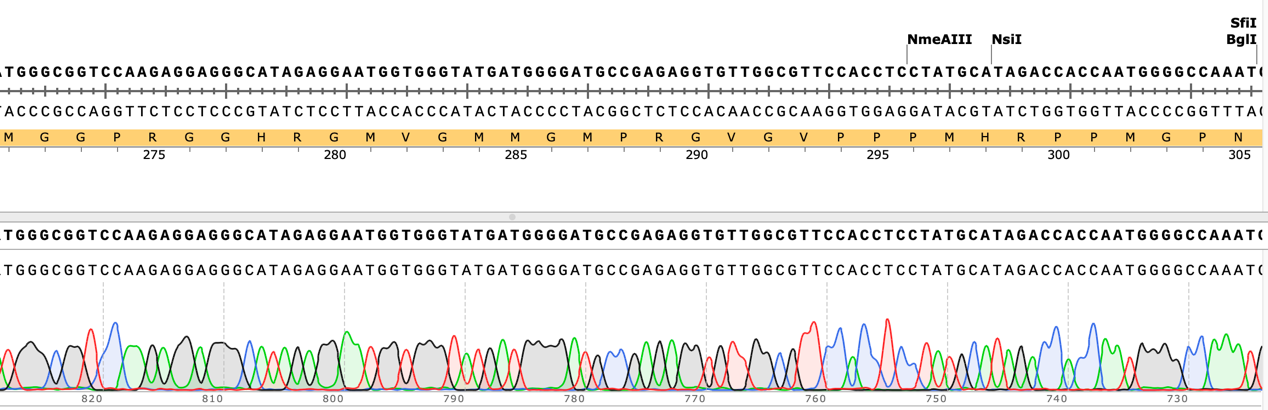


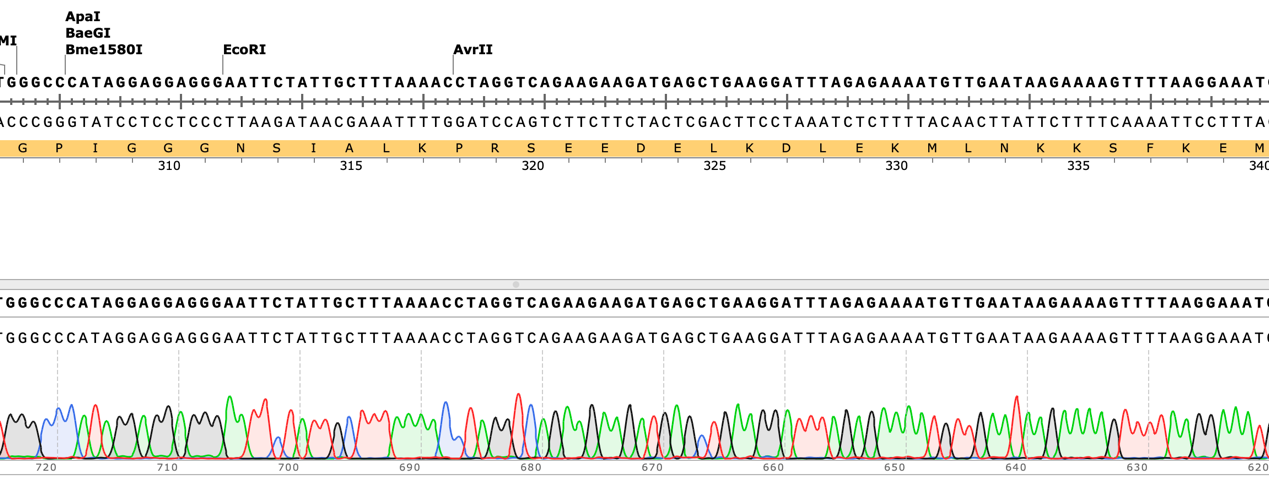


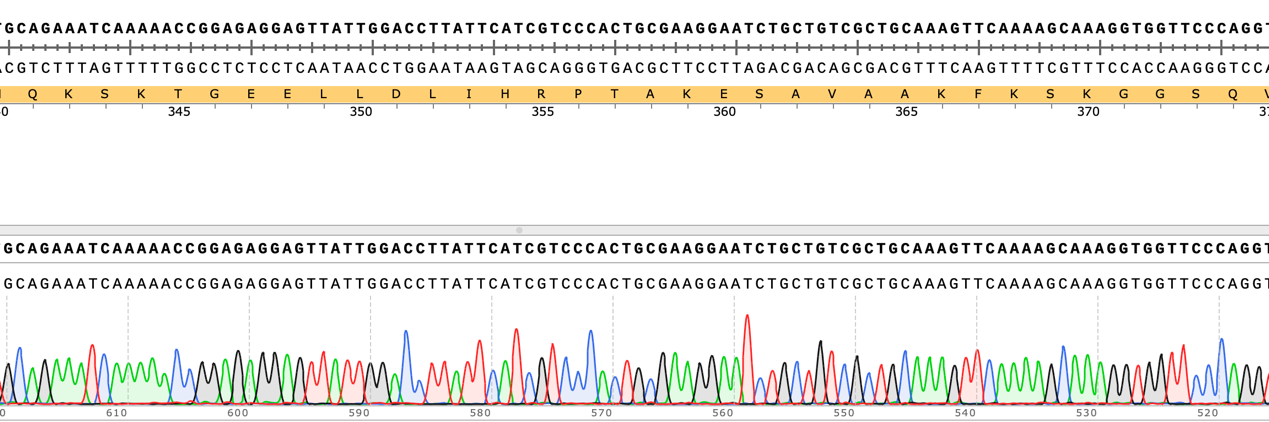


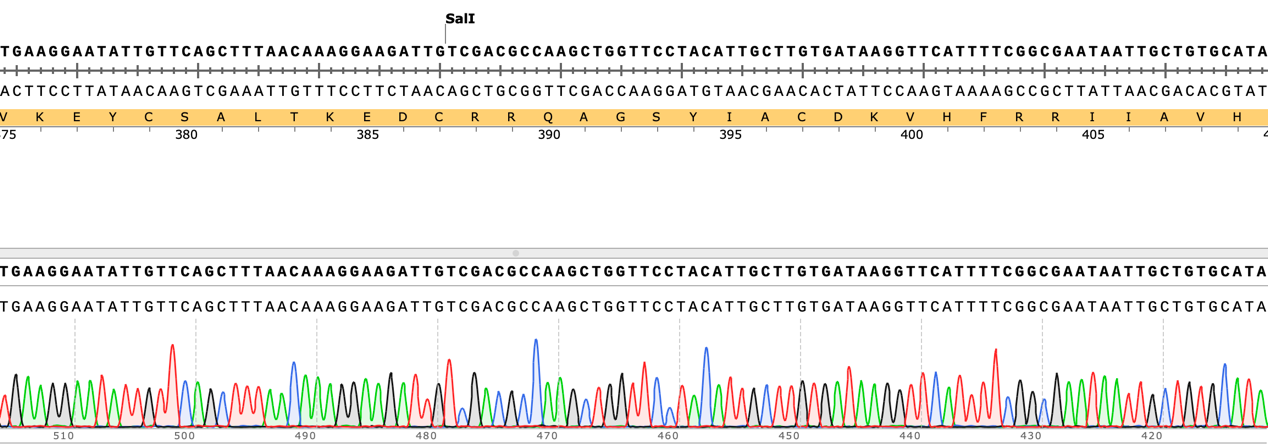


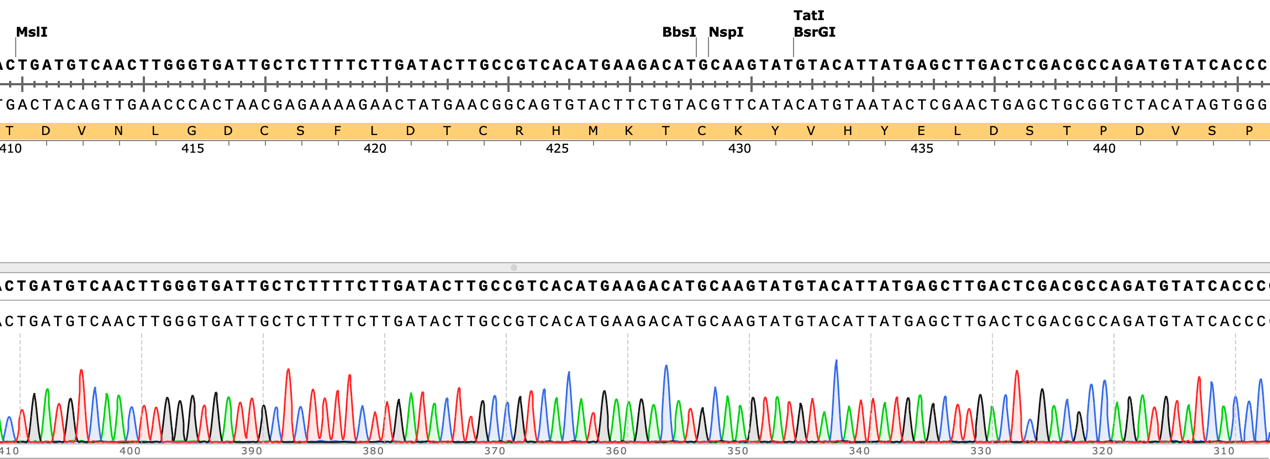


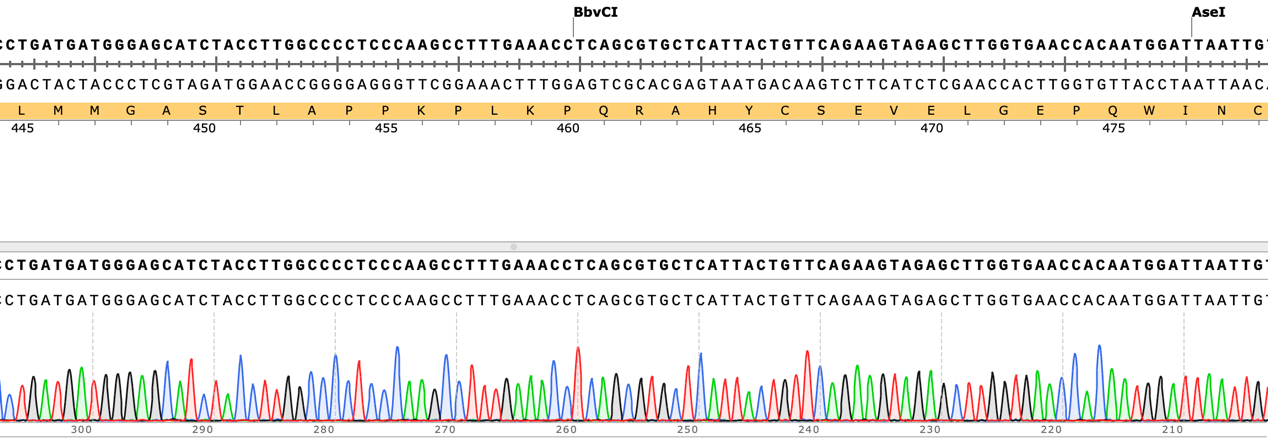


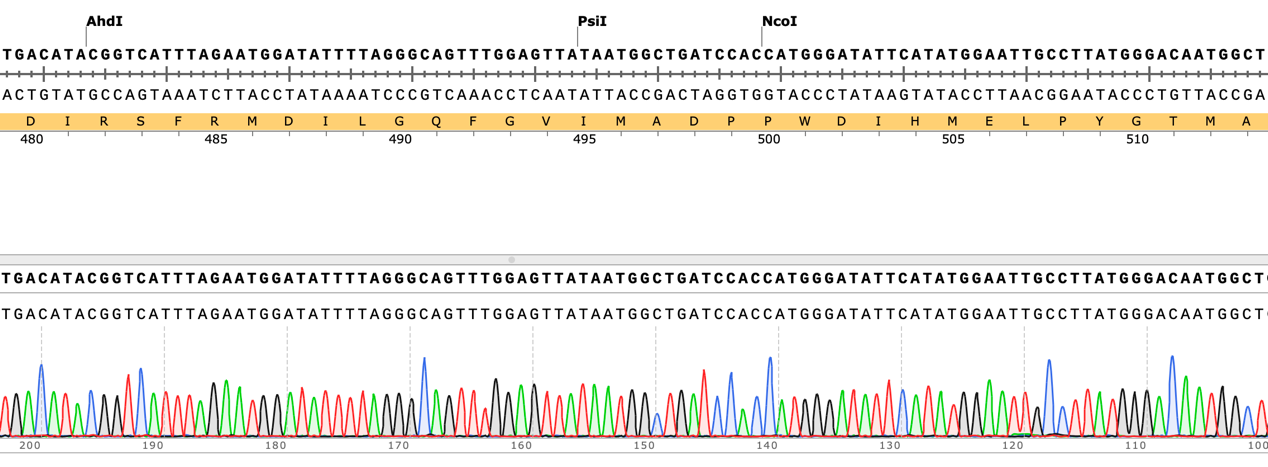


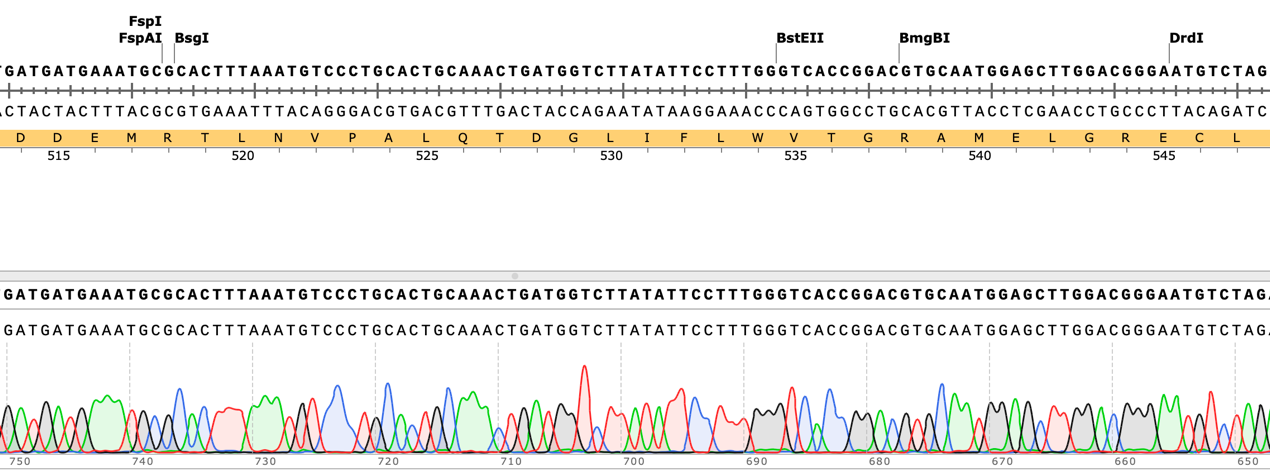


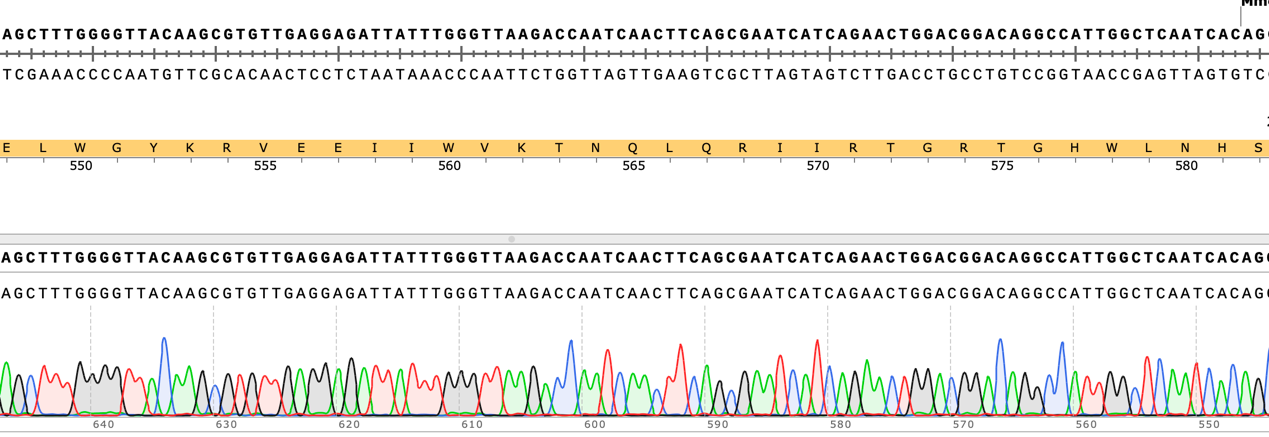


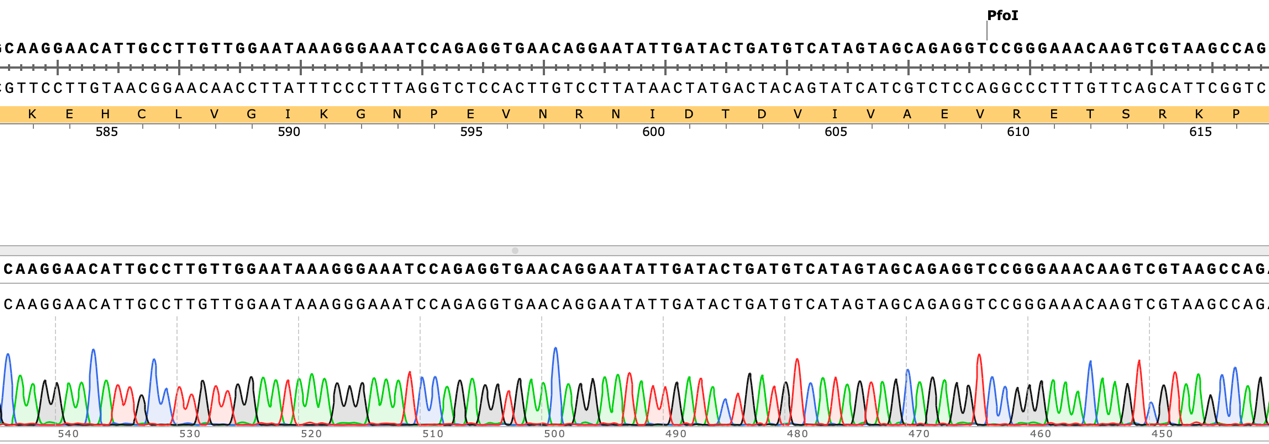


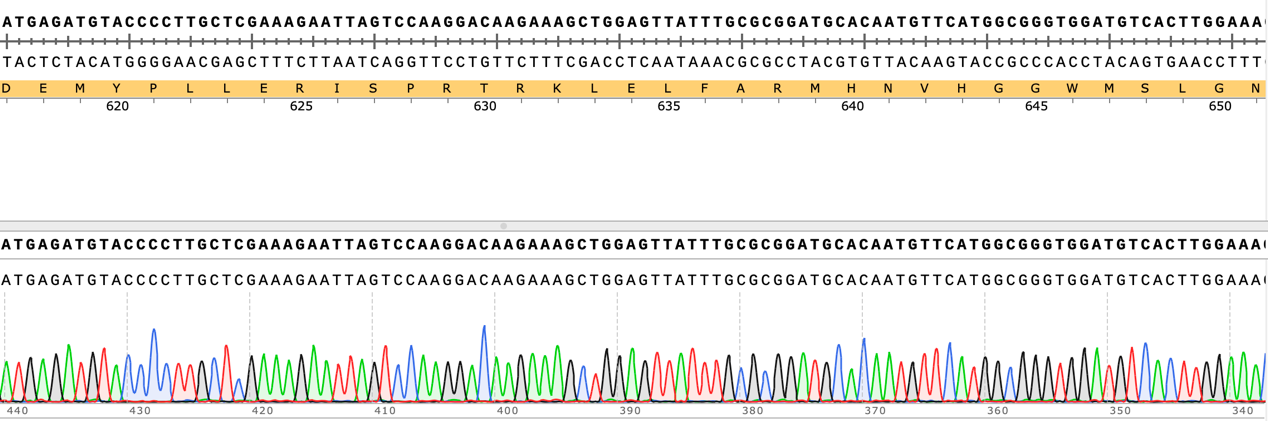


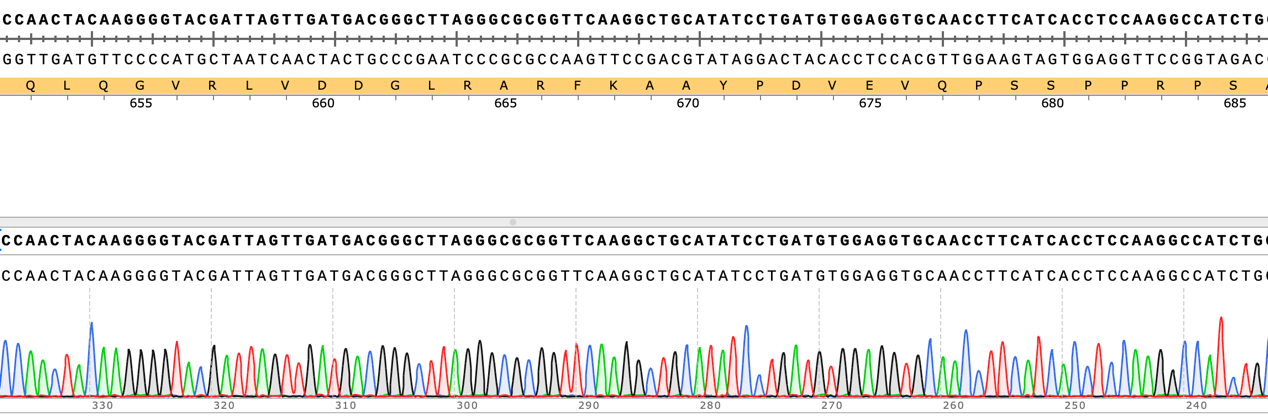


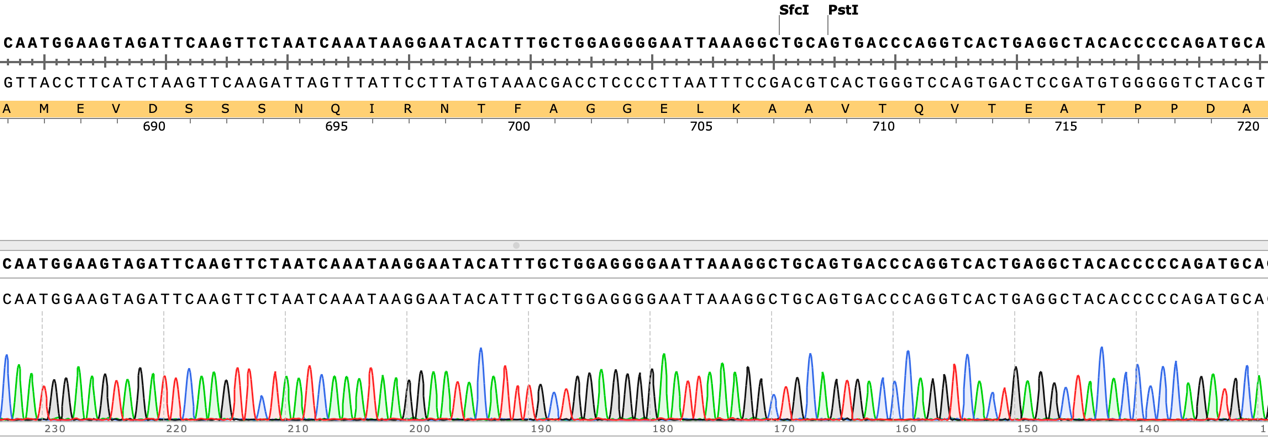


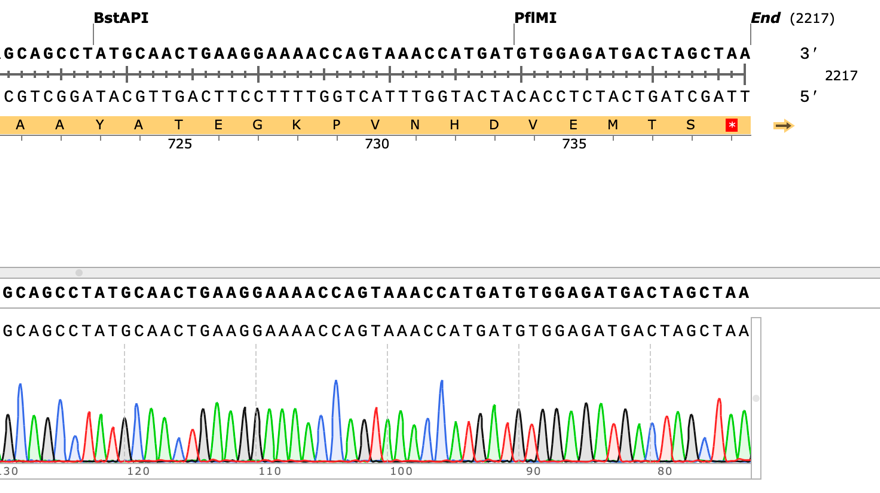


**Figure S4 Expression vector construction of *ToMTA70***

(A) Schematic diagram of vector construction to pMDC32-ToMTA70. (B) Digestion of pDONR207-ToMTA70 plasmid by *Xba* Ⅰ. DNA sizes of marker are shown on the right. The size of *Xba I* digestion fragments are shown on the right. (C) Digestion of pMDC32-ToMTA70 by *Pst* Ⅰ . Fragment sizes of the marker are shown on the right.


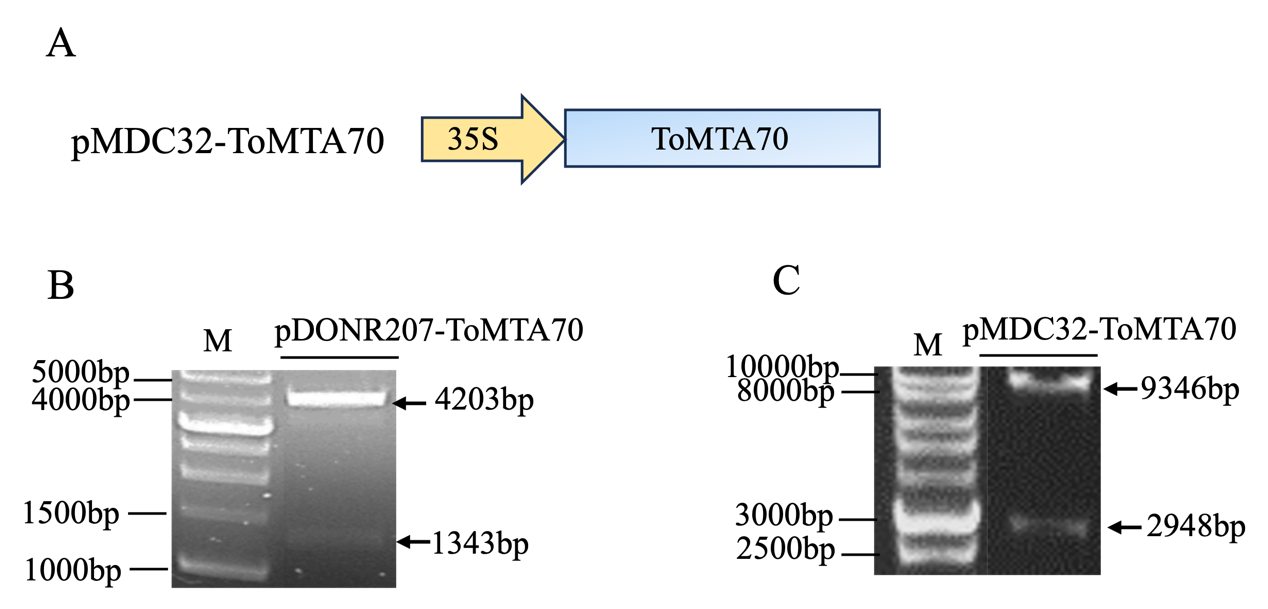


**Figure S5 Prediction of conserved domain and isoelectric point of ToMTA70**

(A)The conserved domain and sequences of m6A methylase MTA70 superfamily in tomato. (B) The molecular weight and isoelectric point of ToMTA70.


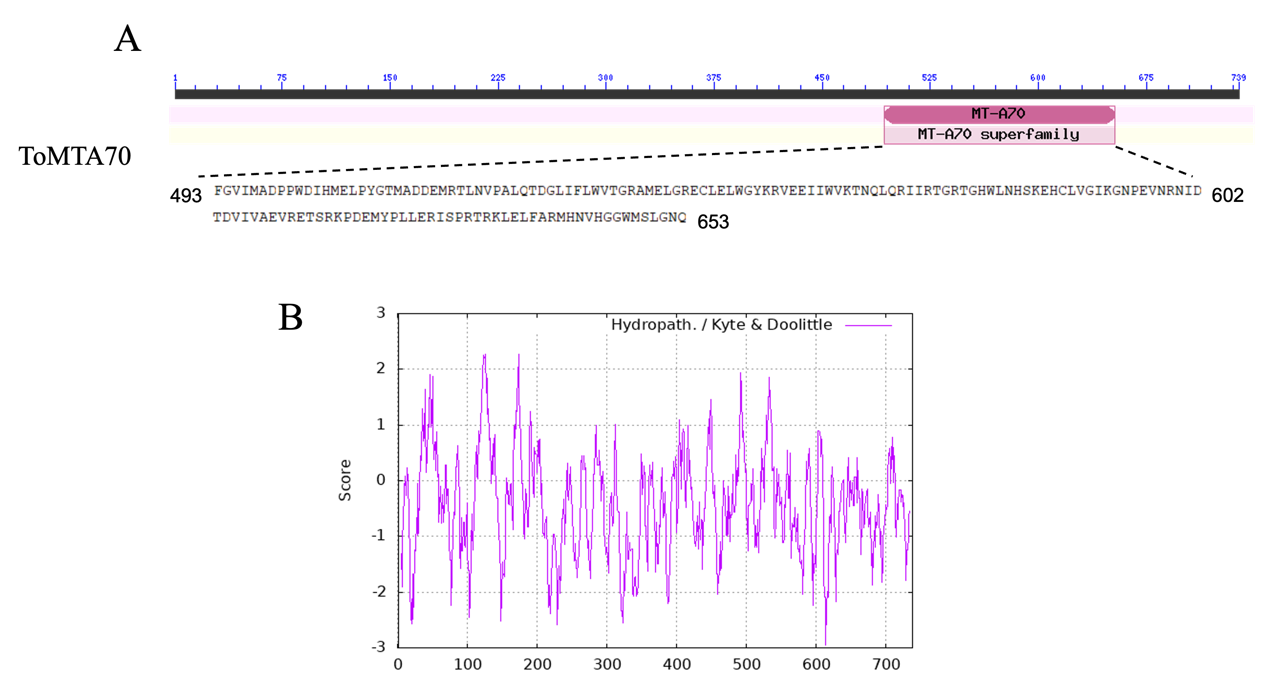


**Figure S6 Amplification and expression vector construction of *ToMTA70-PDS* and *ToMTA70-GUS***

(A) *ToMTA70-PDS* and *ToMTA70-GUS* vector background fragment obtained by PCR with primer pMDC32_PDS-F/R and pMDC32-GUS-F/R in agarose gel.

(B) (C) *ToMTA70-PDS* and *ToMTA70-GUS* insert fragment obtained by PCR with primer 70_PDS-F/R and 70_GUS-F/R in agarose gel.

(D) *ToMTA70-PDS* and *ToMTA70-GUS* fragments were amplified by PCR in *E. coli* DH5a.


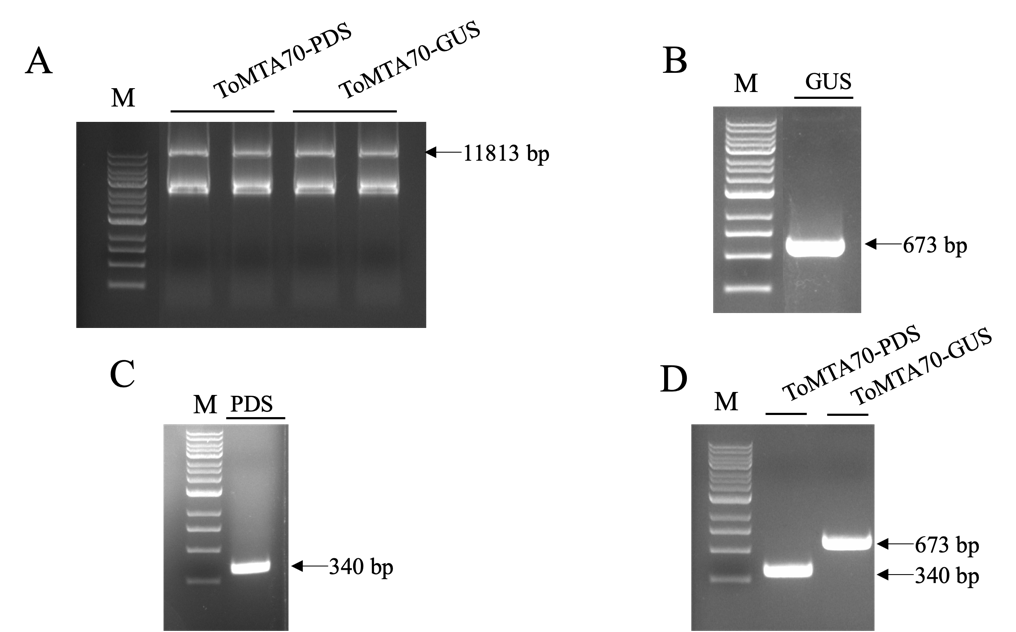


**Figures S7 Sequence map of pMDC32-ToMTA70-PDS**


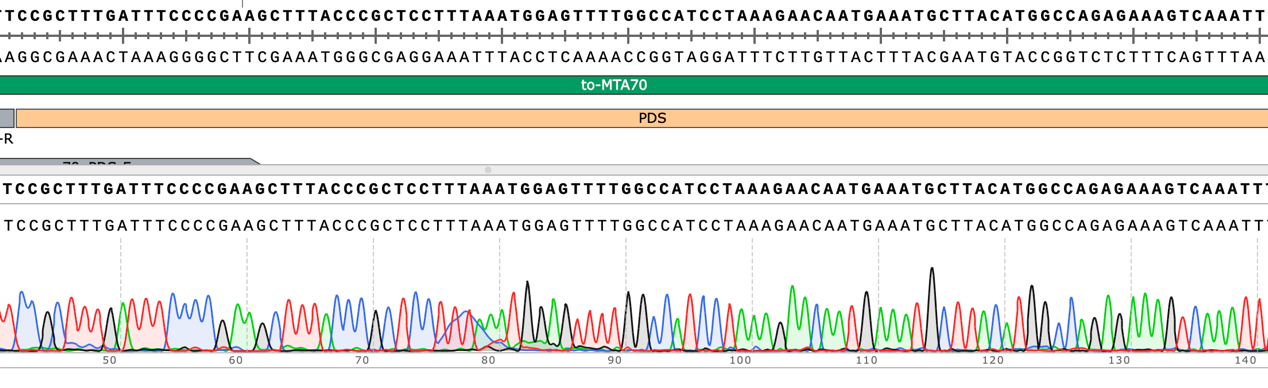


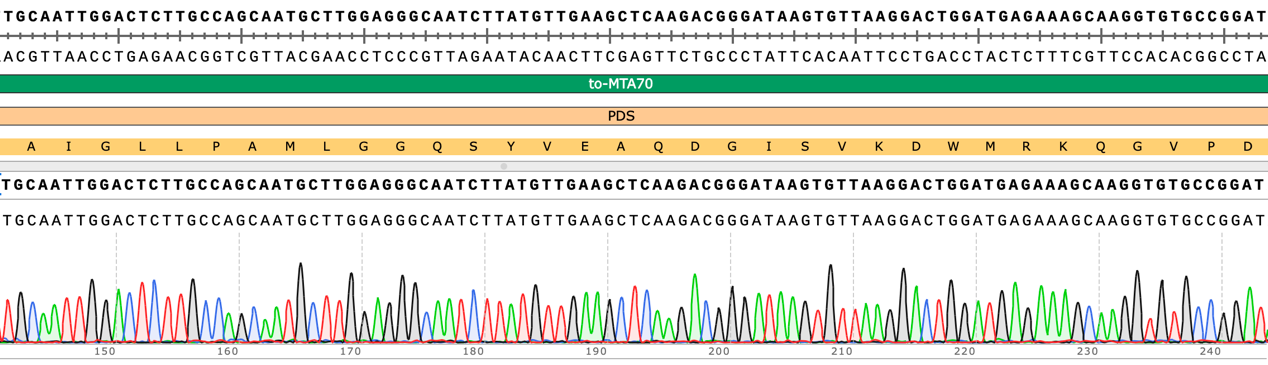


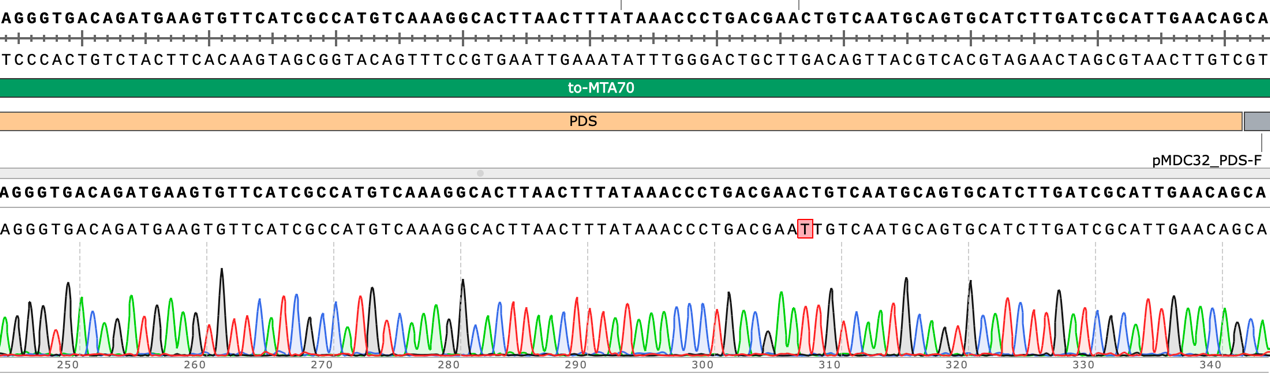


**Figures S8 Sequence map of pMDC32-ToMTA70-GUS**


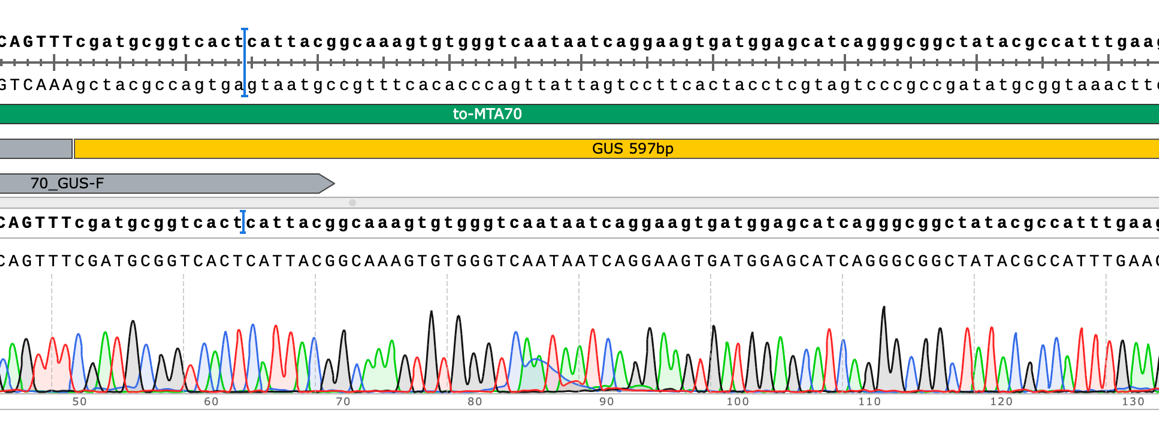


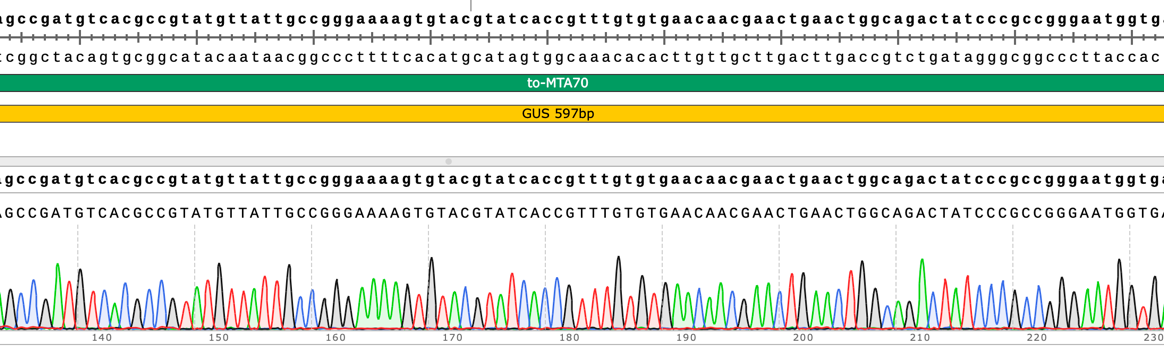


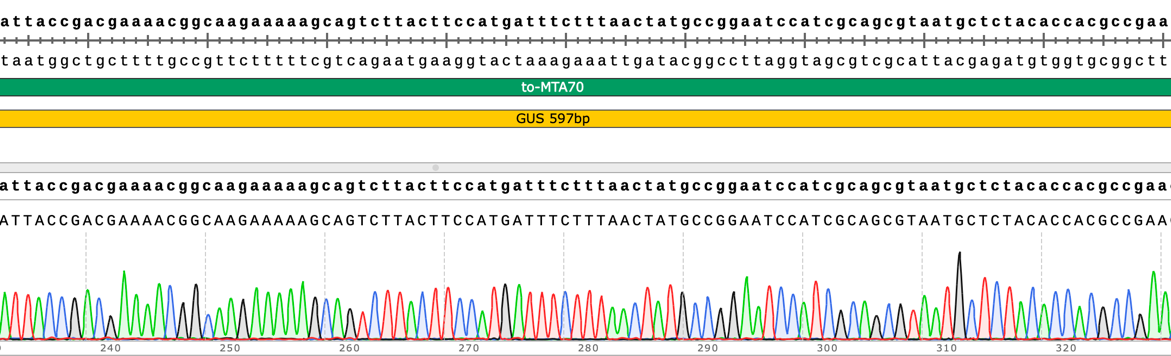


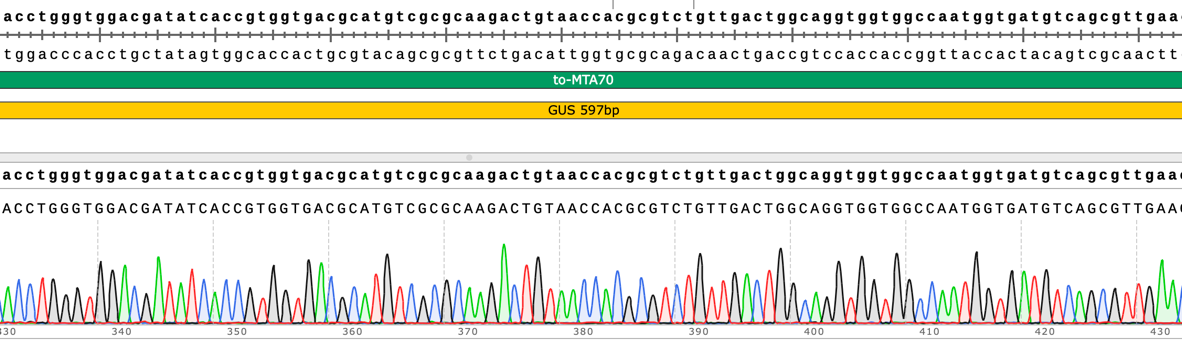


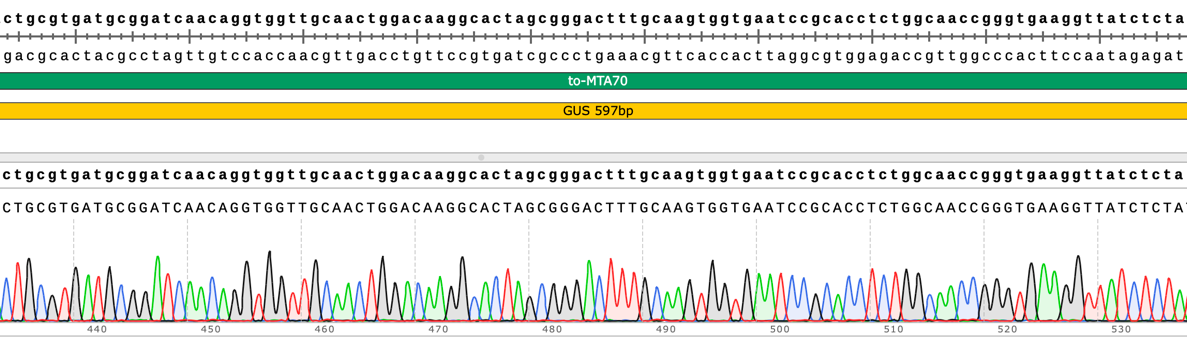


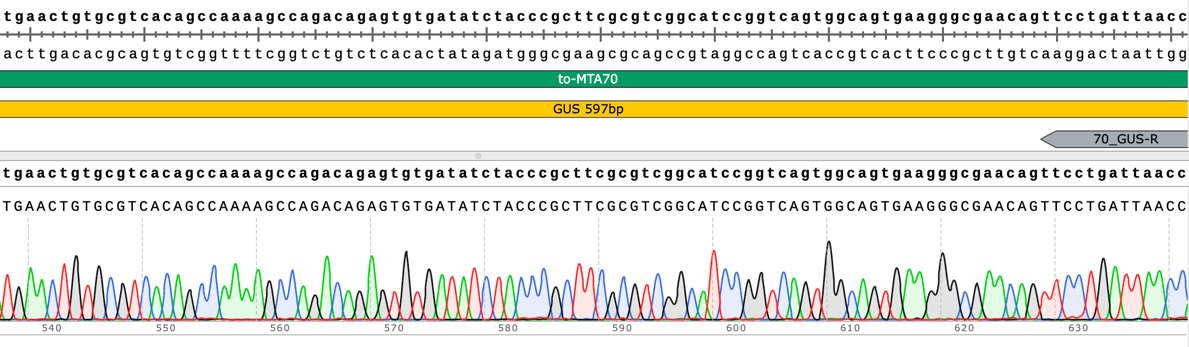


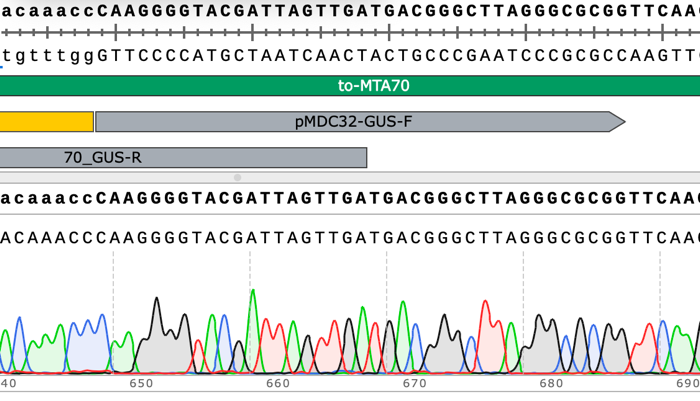

Supplement: Supplementary file 1 [file Data_Sheet_1.docx]
