# Supplementary material for: N6-adenosine-methyltransferase MTA70-like participates antiviral responses in Nicotiana benthamiana
Source: Front Microbiol. 2026 Jan 8;16:1716357. doi: 10.3389/fmicb.2025.1716357 (PMC12826071; doi:10.3389/fmicb.2025.1716357)
Supplement: Supplementary file 2 [file Table_1.docx]

**Table S1 Primers used in this study**

| ID | Sequence (5’-3’) | Use |
| --- | --- | --- |
| ToMTA70_F | GGGGACAAGTTTGTACAAAAAAGCAGGCTTCATGGAAACTCATGCTGACGGC | Cloning |
| ToMTA70_R | GGGGACCACTTTGTACAAGAAAGCTGGGTCTTAGCTAGTCATCTCCACATCATGG |  |
| qPCR-F | TCAAAAGCAAAGGTGGTTCC | RT-qPCR |
| qPCR-R | CCCATCATCAGGGGTGATAC |  |
| NbUBI-F | TCCAGGACAAGGAGGGTATCC | RT-qPCR |
| NbUBI-R | GTCAGCCAAGGTCCTTCCATCC |  |
| 70_GUS-F | TGGATATTTTAGGGCAGTTTCGATGCGGTCACTCATTACG | Cloning |
| 70_GUS-R | TCAACTAATCGTACCCCTTGGGTTTGTGGTTAATCAGGAA |  |
| pMDC32-GUS-F | CAAGGGGTACGATTAGTTGATGACGGGCTTAGGGCGCGG | Cloning |
| pMDC32-GUS-R | AAACTGCCCTAAAATATCCATTCTAAATGACCGTATGTC |  |
| 70_PDS-F | TGGATATTTTAGGGCAGTTTCCGCTTTGATTTCCCCGAAG | Cloning |
| 70_PDS-R | TCAACTAATCGTACCCCTTGCTGTTCAATGCGATCAAGAT |  |
| pMDC32_PDS-F | CAAGGGGTACGATTAGTTGATGACGGGCTTAGGGCGCGG | Cloning |
| pMDC32_PDS-R | AAACTGCCCTAAAATATCCATTCTAAATGACCGTATG |  |

**Table S2 Amino acid composition of ToMTA70 protein**

| Amino acid | TO_MT-A70 | |
| --- | --- | --- |
|  | No. of residues | Percentage of residues |
| Ala (A) | 51 | 6.9% |
| Arg (R) | 47 | 6.4% |
| Asn (N) | 34 | 4.6% |
| Asp (D) | 36 | 4.9% |
| Cys (C) | 13 | 1.8% |
| Gln (Q) | 23 | 3.1% |
| Glu (E) | 59 | 8.0% |
| Gly (G) | 62 | 8.4% |
| His (H) | 22 | 3.0% |
| Ile (I) | 36 | 4.9% |
| Leu (L) | 62 | 8.4% |
| Lys (K) | 41 | 5.6% |
| Met (M) | 35 | 4.7% |
| Phe (F) | 16 | 2.2% |
| Pro (P) | 48 | 6.5% |
| Ser (S) | 50 | 6.8% |
| Thr (T) | 35 | 4.7% |
| Trp (W) | 8 | 1.1% |
| Tyr (Y) | 10 | 1.4% |
| Val (V) | 50 | 6.8% |

**Table S3 Physicochemical parameters of ToMTA70 protein**

| Parameter | ToMTA70 |
| --- | --- |
| Molecular formula | C_3531_H_5687_N_1029_O_1081_S_48_ |
| Molecular weight | 81390.12 |
| Theoretical isoelectric point | 6.37 |
| Instability index | 43.70 |
| Aliphatic index | 78.35 |
| Total number of negatively charged residues (Asp+Glu) | 95 |
| Total number of positively charged residues (Arg + Lys) | 88 |
| Grand average of hydropathicity | -0.429 |

| **Plant** | **Genetic accession number** | **Amino acid sequence homology (%)** |
| --- | --- | --- |
| *Solanum lycopersicum* | XP_004245173.1 | 98.29 |
| *Solanum tuberosum* | XP_006359769.1 | 99.19 |
| *Nicotiana attenuata* | XP_019240291.1 | 89.72 |
| *Capsicum annuum* | XP_016537837.1 | 88.65 |
| *Lycium barbarum* | XP_060208321.1 | 87.30 |
| *Capsicum annuum* | XP_047252722.1 | 83.45 |
| *Ipomoea nil* | XP_019200498.1 | 78.47 |
| *Sesamum indicum* | XP_011101001.2 | 74.73 |
| *Coffea arabica* | XP_027085035.2 | 73.84 |
| *Andrographis paniculata* | XP_051114801.1 | 73.42 |
| *Olea europaea var. sylvestris* | XP_022854353.1 | 73.21 |

**Table S4 Amino acid sequence homology of ToMTA70**
